# Blunting of Colon Contractions in Diabetics with Gastroparesis Quantified by Wireless Motility Capsule Methods

**DOI:** 10.1371/journal.pone.0141183

**Published:** 2015-10-28

**Authors:** Radoslav Coleski, Gregory E. Wilding, John R. Semler, William L. Hasler

**Affiliations:** 1 Department of Internal Medicine, Division of Gastroenterology, University of Michigan Health System, Ann Arbor, Michigan, United States of America; 2 Department of Biostatistics, State University of New York at Buffalo, Buffalo, New York, United States of America; 3 Medtronic, Sunnyvale, California, United States of America; Temple University School of Medicine, UNITED STATES

## Abstract

Generalized gut transit abnormalities are observed in some diabetics with gastroparesis. Relations of gastric emptying abnormalities to colon contractile dysfunction are poorly characterized. We measured colon transit and contractility using wireless motility capsules (WMC) in 41 healthy subjects, 12 diabetics with gastroparesis (defined by gastric retention >5 hours), and 8 diabetics with normal gastric emptying (≤5 hours). Overall numbers of colon contractions >25 mmHg were calculated in all subjects and were correlated with gastric emptying times for diabetics with gastroparesis. Colon transit periods were divided into quartiles by time and contraction numbers were calculated for each quartile to estimate regional colon contractility. Colon transit in diabetics with gastroparesis was prolonged vs. healthy subjects (P<0.0001). Overall numbers of colon contractions in gastroparetics were lower than controls (P = 0.02). Diabetics with normal emptying showed transit and contraction numbers similar to controls. Gastric emptying inversely correlated with overall contraction numbers in gastroparetics (r = -0.49). Numbers of contractions increased from the 1^st^ to 4^th^ colon transit quartile in controls and diabetics with normal emptying (P≤0.04), but not gastroparetics. Numbers of contractions in the 3^rd^ and 4^th^ quartiles were reduced in gastroparetics vs. healthy controls (P≤0.05) and in the 4^th^ quartile vs. diabetics with normal emptying (P = 0.02). Numbers of contractions were greatest in the final 15 minutes of transit, but were reduced in gastroparetics vs. healthy controls and diabetics with normal emptying (P≤0.005). On multivariate analyses, differences in numbers of contractions were not explained by demographic or clinical variables. In conclusion, diabetics with gastroparesis exhibit delayed colon transit associated with reductions in contractions that are prominently blunted in latter transit phases and which correlate with delayed gastric emptying, while diabetics with normal emptying show no significant colonic impairments. These findings emphasize diabetic gastroparesis may be part of a generalized dysmotility syndrome.

## Introduction

Patients with long-standing diabetes mellitus experience a range of symptoms involving different regions of the gastrointestinal tract and show evidence of sensorimotor dysfunction in any gut segment [1]. Gastroparesis is the most extensively studied luminal complication of diabetes and presents with varying degrees of nausea, vomiting, bloating, early satiety, fullness, and pain. Delays in gastric emptying most often are characterized using scintigraphy and are reported in 27–58% of type 1 diabetics and up to 30% with type 2 disease [2–4]. Increases in constipation with diabetes are inconsistently reported, with some studies observing prevalences as high as 60% while others noting no increase relative to non-diabetic populations [5–10]. Delays in colon transit have sometimes been reported in diabetics with constipation using radiopaque marker or scintigraphy techniques [11–15].

Adoption of wireless motility capsule (WMC) testing, investigated both for gastroparesis and constipation, has permitted routine quantification of transit in all gut regions in a single test [16–20]. Most other diagnostic modalities are specific for only a single gastrointestinal region. Retrospective case series employing WMC techniques have uncovered significant numbers of patients with presumed idiopathic or diabetic gastroparesis who also exhibit abnormal small intestinal and/or colonic transit, emphasizing the existence of unsuspected generalized dysmotility [21, 22]. Likewise in a prospective WMC investigation, some diabetics with gastroparesis exhibited prolongation of colon transit [20].

WMC tests additionally acquire luminal pressure data that quantify numbers of contractions in different gut regions [21]. Region specific differences in contractility within the colon can be estimated by dividing the colon transit period into quartiles by time [23]. WMC pressure recordings have documented reduced gastric contractions with severe diabetic gastroparesis, as well as increases in colon contractility in constipation especially with associated irritable bowel syndrome [23, 24]. The relation of delayed gastric emptying to regional colon contractions in diabetics with gastroparesis is uninvestigated.

This investigation analyzed WMC and clinical data files from a published parent study of WMC testing in gastroparesis to test the hypotheses that: (i) diabetics with gastroparesis exhibit blunted numbers of colon contractions in association with delayed colonic transit compared to healthy controls, whereas diabetic patients with normal gastric emptying do not show significant reductions, (ii) reductions in colon contractions in diabetics with gastroparesis are most prominent in the latter phases of colon transit, and (iii) colon contractile impairments associated with diabetic gastroparesis do not relate to other demographic or clinical factors including age, sex, and body mass index [16]. Through these analyses, we characterized colon motor dysfunction associated with diabetic gastroparesis verifying the presence of generalized gut dysfunction in this condition and thereby providing a physiologic foundation for future clinical investigations into the pathogenesis and management of diabetic constipation in patients with associated gastroparesis.

## Materials and Methods

### Human Subject Data

WMC data files and clinical and demographic information from 20 type 1 or type 2 diabetics (5 men, 15 women, age 46.5±2.5 years) from seven centers of a parent investigation (ClinicalTrials.gov Identifier: NCT001282884) of capsule quantification of gastric emptying times were analyzed for this study [16]. All diabetics reported nausea, vomiting, early satiety, bloating, and/or epigastric discomfort on the Mayo Clinic GI Disease Questionnaire for ≥6 months and had been previously diagnosed with gastroparesis based on scintigraphy performed prior to recruitment [25]. Exclusion criteria from the parent study were extreme gastric stasis on prior scintigraphy (>90% gastric retention at 2 hours), gastric bezoar, peptic stricture or ulcer disease, dysphagia, prior gastrointestinal surgery (except appendectomy or cholecystectomy), uncontrolled diabetes (hemoglobin A1c >10%), weight loss >10 pounds over the previous 2 months, vomiting >1 episode per day, and abdominal pain requiring daily narcotics. Constipation was not an inclusion criterion; individuals with self-reported defecation frequencies <3/week were excluded.

WMC data files and clinical and demographic information from 41 healthy volunteers (26 men, 15 women, age 31.9±1.8 years) recruited into the parent study were analyzed for this investigation. Healthy subjects reported no gastrointestinal symptoms upon screening with the Mayo Clinic GI Disease Screening Questionnaire and had no prior gastrointestinal surgery, were not morbidly obese (body mass index <35 kg/m^2^), were not on medications that affect gastrointestinal transit, and reported no cardiovascular, endocrine, renal, or hepatic disease.

The analyses conducted for this investigation were approved by the University of Michigan Health System Institutional Review Board (IRBMED), which granted a Waiver of Informed Consent to access the WMC data and all original investigations were conducted in accordance with the principles expressed in the Declaration of Helsinki.

Proton pump inhibitors were stopped 7 days, histamine_2_ antagonists were stopped 2 days, and antacids were stopped one day prior to study. Prokinetic medications (metoclopramide, erythromycin, domperidone, tegaserod) and selected antiemetics (muscarinic and 5-HT_3_ antagonists) were held for 2 days and opiates and non-steroidal anti-inflammatory agents were stopped 7 days before study. Tobacco products were not permitted for eight hours before and for 8 hours after WMC ingestion. No ethanol consumption was permitted for 24 hours prior and 72 hours after WMC ingestion. Strenuous activities and >15 minutes per day of aerobic activity were prohibited 72 hours after WMC ingestion. Urine pregnancy testing was performed on women of child-bearing potential before testing. Diabetics injected half of their usual morning insulin dose before testing to minimize the risk of hypoglycemia.

On the day of study, the WMC was swallowed followed by consumption of a low-fat egg substitute meal as described in the Supplemental Information section (S1 File). Concurrent WMC and scintigraphic testing was performed according to standardized methods [16].

### WMC Data Analyses

#### Quantification of Luminal pH and Temperature Parameters

Digitized pH and temperature data were downloaded from the WMC data receiver to a laptop computer (Dell Latitude, Dell Computer Corporation, Round Rock, TX) for analysis (MotilitGI, SmartPill Corporation, Buffalo, NY). The beginning of the pH recording was defined when the WMC was swallowed. WMC passage into the duodenum was defined when the pH abruptly rose ≥2 pH units from the lowest postprandial value to at least 4 and did not decrease below 4 for >10 minutes at any subsequent time during the recording [26]. The WMC gastric emptying time in hours was calculated by subtracting the time of capsule ingestion from the time the capsule passed into the duodenum. For the analyses of this investigation, diabetics were stratified into those with gastroparesis (defined by WMC gastric emptying time >5 hours) and individuals with normal gastric emptying (WMC gastric emptying time ≤5 hours). WMC passage across the ileocecal junction was detected when the pH abruptly decreased by ≥1.0 pH unit at least 30 minutes after gastric evacuation and persisted for at least 10 minutes [26]. This pH decrease is observed in >85% of healthy subjects and constipated subjects and has been validated in studies employing concurrent whole gut scintigraphy [17, 27]. Temperature was consistent from 96.0 to 99.5°F throughout the gut. Anal capsule expulsion was determined by abrupt 0.045°F per second temperature decreases. WMC colon transit times were calculated from the time of capsule ileocecal passage to the time the capsule was expelled across the anus and were compared in healthy controls, diabetics with gastroparesis, and diabetics with normal gastric emptying by WMC [26]. Correlation coefficients (r values) were calculated to correlate fasting blood glucose levels from the morning of study in the diabetic patients to colon transit to ascertain the influence of acute glycemia.

#### Quantification of Luminal Contractions

Motor activity from the pressure recordings was analyzed from the times of ileocecal passage to anal expulsion using standardized software (GIMS Data Viewer, Release 1.8, SmartPill Corporation, Buffalo, NY). Numbers of contractions >25 mmHg in amplitude were calculated per 15 minutes of recording time to facilitate comparisons for WMC recording periods of varying length in patients with different colon transit times. Correlation coefficients (r values) were computed relating overall numbers of colon contractions with WMC gastric emptying times selectively in the diabetics with gastroparesis. Correlation coefficients also were calculated to correlate fasting blood glucose levels from the study day in the diabetic patients to overall number of contractions to assess if any contractile abnormalities related to acute glycemic control. The total colon transit time was divided by four and the first, second, third, and fourth quartile transit times were determined. Numbers of contractions >25 mmHg in amplitude per 15 minutes were quantified for each quartile to estimate regional colon activity using previously published and validated methods [23]. Numbers of contractions from the final 15 minutes of transit were separately analyzed to estimate distal contractile activity associated with defecation. Overall numbers of contractions, numbers of contractions in each of the 4 colon transit quartiles, and numbers of transit in the final 15 minutes of colon transit prior to WMC expulsion were compared in healthy volunteers, diabetics with gastroparesis defined by delayed WMC gastric emptying, and diabetics with normal gastric emptying.

Data from 25 healthy volunteers and 3 diabetics from a prior WMC study of regional gut transit were excluded from the analyses of this investigation [20]. Five healthy subjects exhibited abrupt WMC evacuation from the stomach prior to meal completion that precluded accurate determination of WMC gastric emptying times. Twenty healthy subjects and 3 diabetics from the prior paper exhibited loss of pressure data for >5% of recording time during the period of WMC colon transit that precluded accurate calculation of numbers of colon contractions [20]. Signal dropout causing this data loss usually occurred when the receiver was inadvertently placed too far away from the subject during sleep.

Demographic and clinical information from healthy volunteers, diabetics with gastroparesis, and diabetics with normal gastric emptying were included in multivariate analyses to verify that differences in pressure parameters were secondary to transit time block or subject group only. Demographic variables from all three groups included age, sex, and body mass index (BMI). Variables specific for diabetics with gastroparesis and diabetics with normal gastric emptying included fasting blood glucose on the study day and routine use of insulin, oral hypoglycemic agents, and antidepressant medications.

### Statistical Analyses

All results were expressed as mean ± SEM. To describe the observed variability in the contractile data across colon transit quartiles and to test for differences between groups, a multivariate linear model was fit to each considered endpoint. Each endpoint was fit as a function of group, time, and a group by time interaction. To account for the within-group dependence structure, the model assumed that the distribution of the error terms for each subject to be multivariate normal with zero mean and an unstructured covariance structure. Once a model was fit, specific linear contrasts based on the estimated model parameters were constructed and used to test hypotheses of interest [28]. Differences in contractions during the final 15 minutes of colon transit were assessed using a single factor analysis of variance (ANOVA) model. All tests were two-sided and tested at a 0.05 nominal significance level and standard diagnostic plots were used to assess model fit. The ln(X + 1) transformation was used in all models in order to meet statistical assumptions. Single factor ANOVA was performed to determine if there were significant differences in colon transit between healthy volunteers, diabetics with gastroparesis, and diabetics with normal gastric emptying. All statistical analyses were carried out using SAS version 9.1.3 statistical software (Cary, NC).

## Results

### Subject Characteristics

Baseline demographic and clinical characteristics of individuals in the three subject groups are shown in Table 1. There were significant age differences between groups (P<0.0001); healthy controls were youngest while diabetic patients with normal gastric emptying were oldest. Likewise, there were differences in sex distribution between groups (P = 0.01); both diabetic groups were predominantly women while healthy volunteers were predominantly men. However, there were no differences in sex distribution between diabetics with normal vs. delayed gastric emptying (P = 0.60). There were no differences in BMI between subject groups (P = 0.20).

**Table 1:** Subject Characteristics for Each of the Three Groups.

Characteristic	Healthy Volunteers	Diabetic Patients with Normal Emptying	Diabetic Patients with Gastroparesis	P Value
Age (years)(mean±SEM)	31.9±1.8	53.9±8.0	41.6±3.1	<0.0001
Sex (M/F)(% female)	26/15 (36.6%)	1/7 (87.5%)	4/8 (66.7%)	0.01
BMI (kg/m^2^)(mean±SEM)	30.0±0.8	28.8±2.1	27.8±1,5	0.20

### Representative WMC Recordings

**Fig 1:**
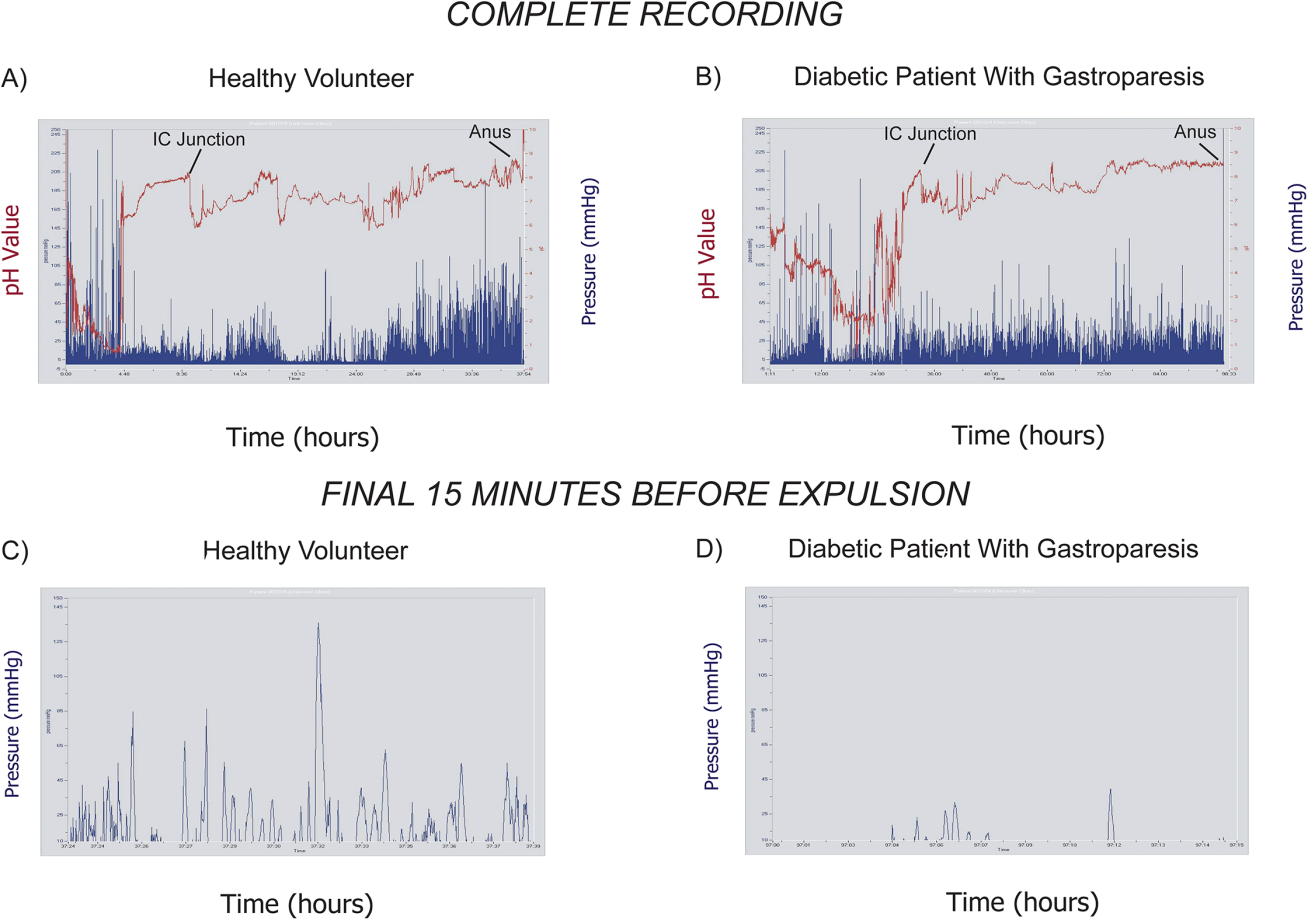
Representative WMC Recordings. Representative WMC recordings are shown from a healthy volunteer (A, C) and a diabetic with gastroparesis (B, D). In the complete recording from the healthy subject, there is an abrupt pH decrease (red) at 10 hours and 15 minutes reflecting ileocecal (IC) transit (A). The capsule is expelled at 37 hours and 39 minutes. In the diabetic, IC transit occurs at 33 hours and 15 minutes and the capsule is expelled at 97 hours and 14 minutes (B). Pressure activity (blue) increases prior to WMC expulsion in the healthy subject, but not in the diabetic with gastroparesis. In the final 15 minutes of colon transit, contractions (blue) are frequent and intense in the healthy volunteer (C) but are reduced in the diabetic (D).

Representative WMC recordings are shown from a healthy volunteer and a diabetic patient with gastroparesis (WMC gastric emptying time >5 hours). In the pH tracing (red) from the healthy subject, subtracting times of ileocecal transit (10:15) from anal expulsion (37:39) results in a colon transit time of 27 hours and 24 minutes (Fig 1A). During the period of colon transit, contractions (blue) were variable but increased prior to WMC expulsion. In the pH recording from the diabetic patient with gastroparesis (red)(WMC gastric emptying time 29:45), colonic transit (63:59) was calculated by subtracting ileocecal passage (33:15) from anal expulsion (97:14)(Fig 1B). In contrast to the healthy subject, contractions (blue) did not increase before the WMC was anally expelled. Pressure activity significantly increased in the 15 minutes before WMC expulsion from the anus in the healthy volunteer, but decreased in this latter phase of colon transit in the diabetic patient. Expanded views of the final 15 minutes (blue) show frequent contractions in the healthy subject compared to the infrequent motor activity in the individual with diabetic gastroparesis (Fig 1C and 1D).

### Colon Transit Time in Relation to WMC Gastric Emptying Times

**Fig 2:**
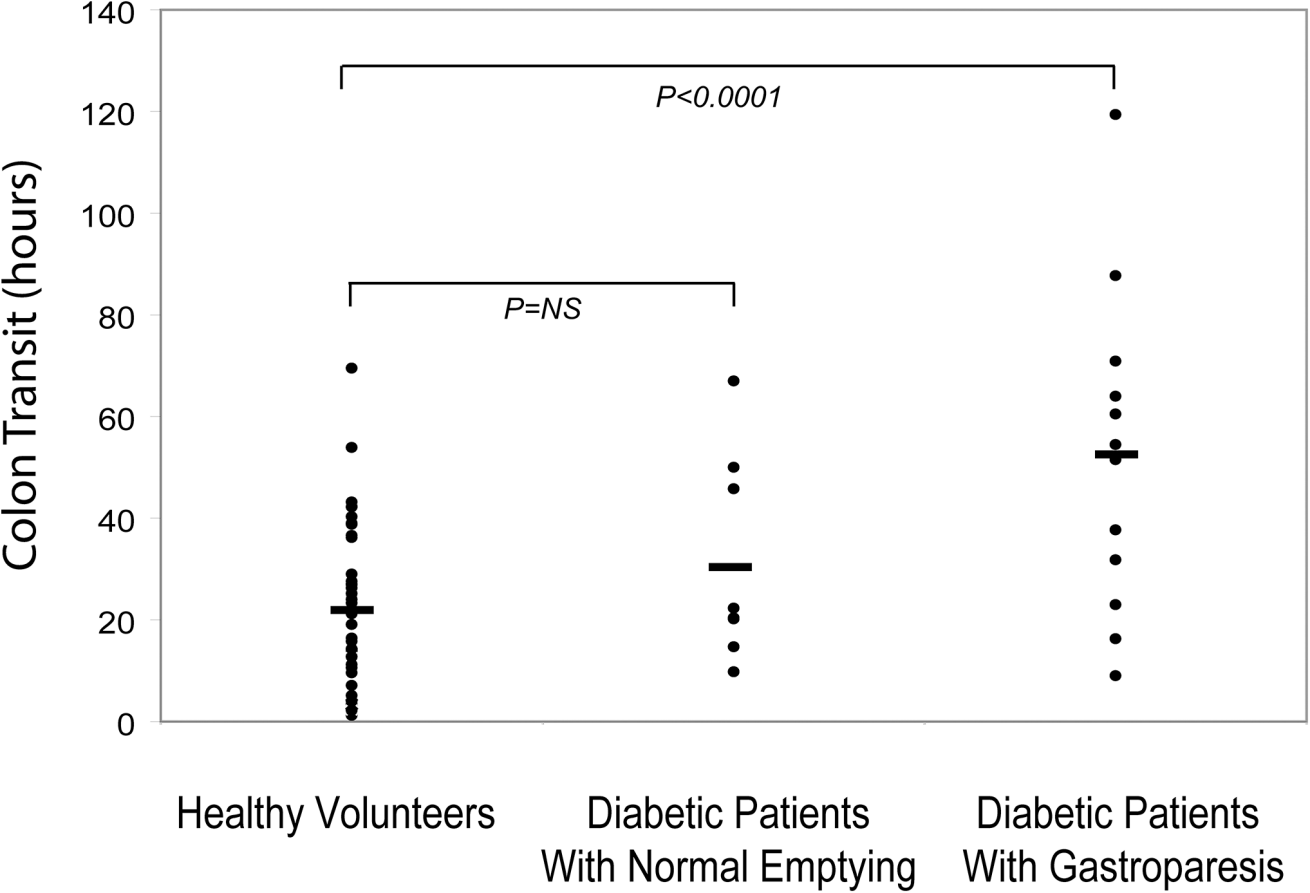
Colon Transit Times in Relation to Gastric Emptying. Colon transit times are compared in individual healthy volunteers, diabetics with normal gastric emptying, and diabetics with gastroparesis. Gastroparesis patients exhibited significant prolongation of colon transit versus healthy subjects (P<0.0001), while diabetics with normal gastric emptying showed colon transit times that were not different from healthy controls (P = NS).

Colon transit times were compared in diabetics with gastroparesis, diabetics with normal gastric emptying, and healthy volunteers. Diabetic gastroparetic patients exhibited delayed colon transit versus healthy volunteers (P<0.0001)(Fig 2). Conversely, diabetic patients with normal gastric emptying showed no significant differences in colon transit times versus the healthy subjects or diabetics with gastroparesis (P = NS). Colon transit times in diabetics with gastroparesis and with normal gastric emptying did not correlate with fasting blood glucose values obtained on the study day (r = 0.09).

### Differences in Overall Numbers of Colon Contractions

**Fig 3:**
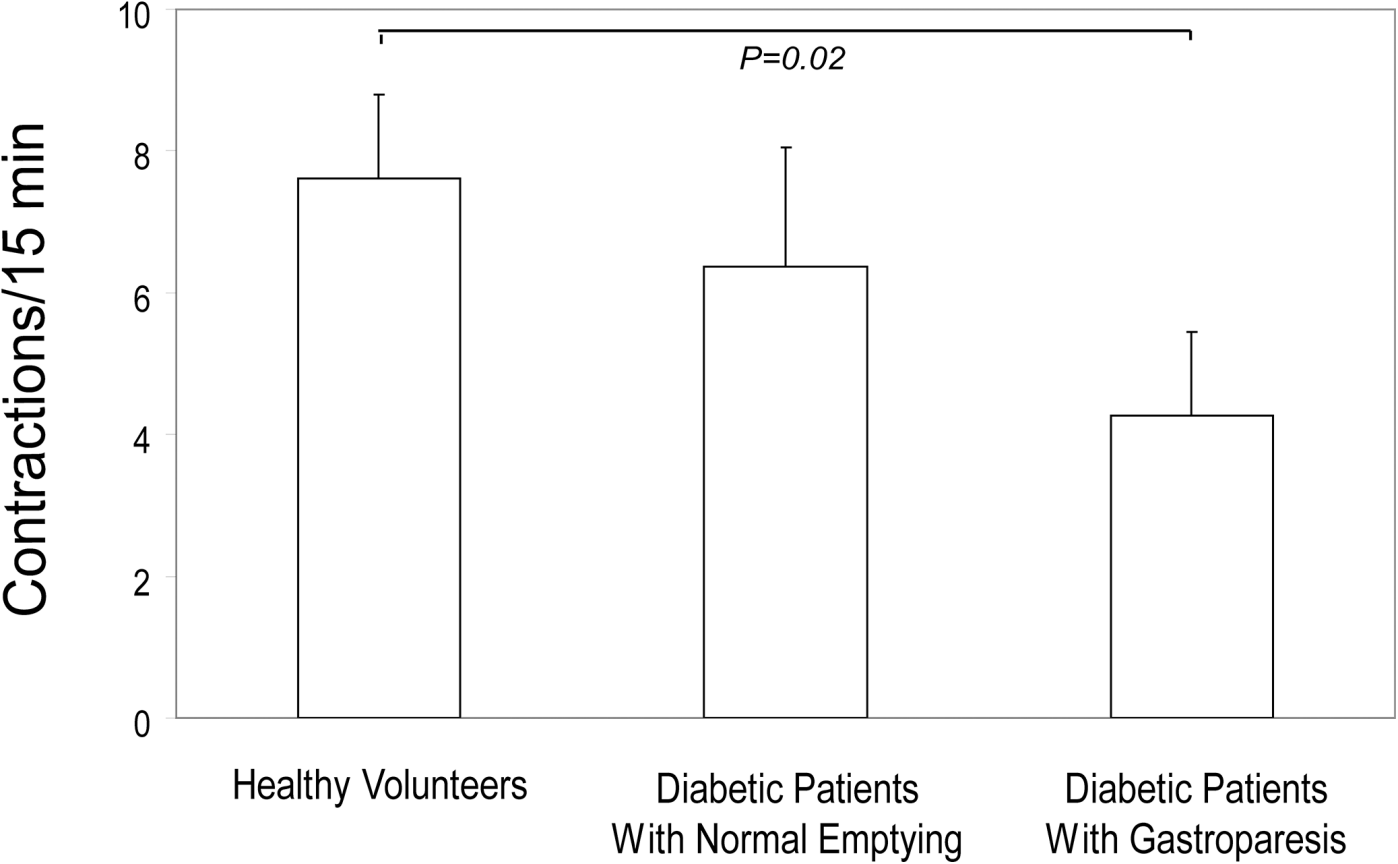
Numbers of Colon Contractions. Overall numbers of colon contractions are plotted for the three groups. Diabetics with gastroparesis showed reductions in overall numbers of contractions compared to healthy subjects (P = 0.02). Overall contractions in diabetics with normal gastric emptying were not different than in diabetic patients with gastroparesis or in healthy controls (P = NS).

**Fig 4:**
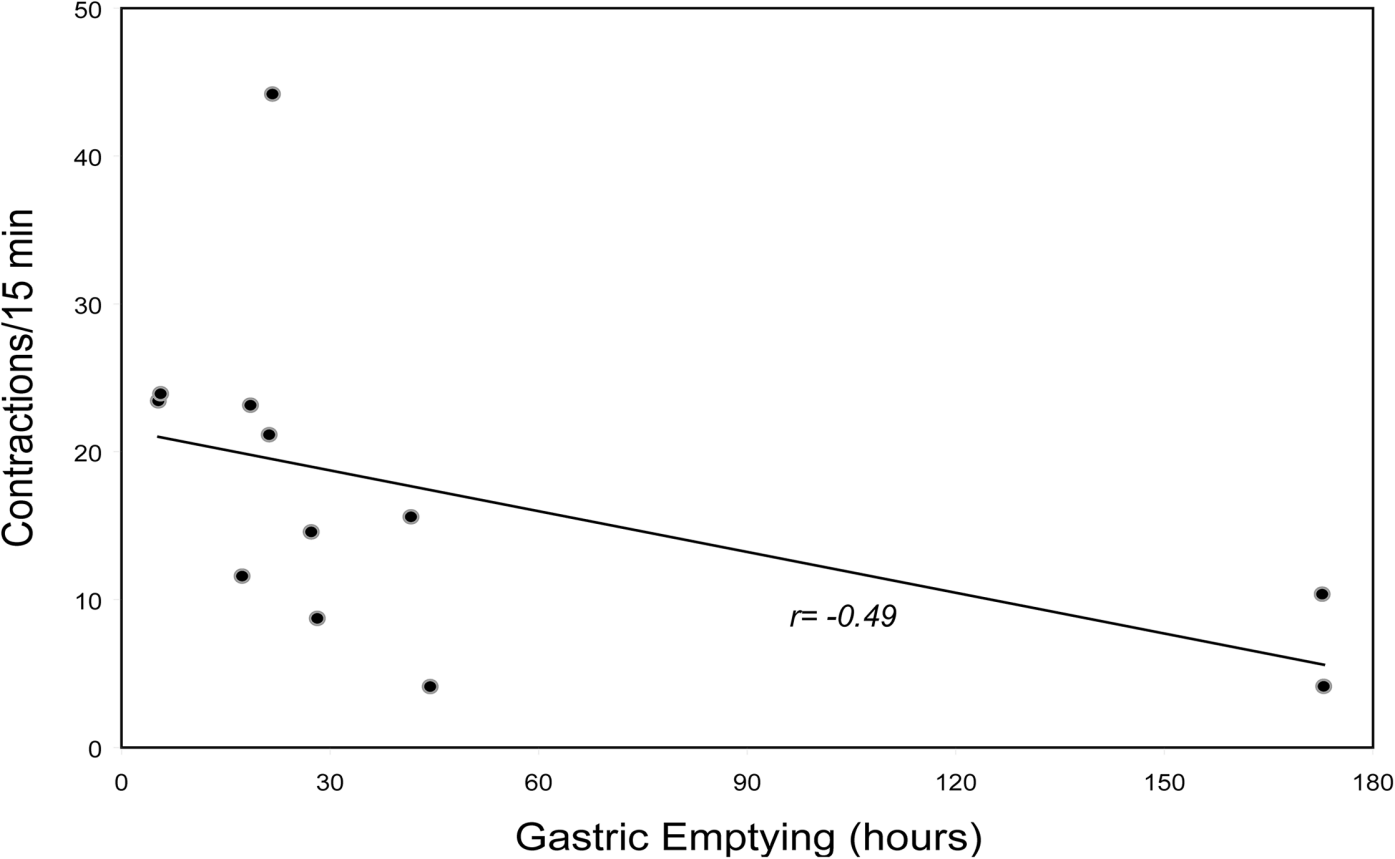
Correlation of Colon Contraction Numbers with Gastric Emptying. The correlation of overall numbers of colon contractions with gastric emptying times is plotted for diabetics with gastroparesis. There was a moderately good inverse correlation (r value -0.49) with patients with the most severe emptying delays exhibiting lower numbers of contractions.

Numbers of contractions over the entire colon transit period were compared. Overall contractions were reduced in diabetics with gastroparesis versus healthy subjects (P = 0.02)(Fig 3). In contrast, overall numbers of contractions in diabetics with normal gastric emptying were not different from either the healthy volunteers or diabetics with gastroparesis (P = NS). Overall numbers of colon contractions correlated inversely with WMC gastric emptying times selectively in the diabetics with gastroparesis with a correlation coefficient (r value) of -0.49 (Fig 4). Fasting glucose levels were not different in diabetics with gastroparesis (131±16 mg/dL) versus with normal emptying (126±13 mg/dL)(P = NS) and overall numbers of colon contractions did not correlate with fasting glucose levels obtained on the morning of study (r = 0.10).

### Regional Differences in Numbers of Colon Contractions

**Fig 5:**
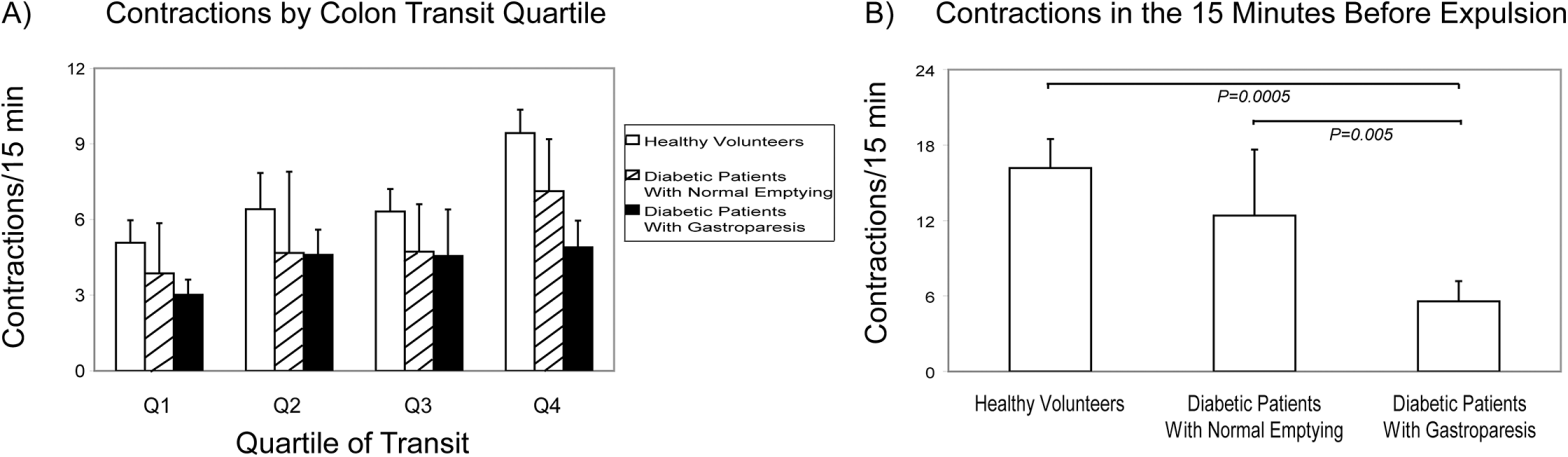
Regional Differences in Numbers of Colon Contractions. Numbers of colon contractions are plotted as a function of colon transit quartile (A) and for the final 15 minutes of colon transit (B). Healthy volunteers (P<0.0001) and diabetics with normal gastric emptying (P = 0.04) showed increased numbers of contractions from the first to fourth quartiles, while contractions did not increase from the first to fourth quartiles in diabetics with gastroparesis (P = NS). Compared to healthy controls, diabetics with gastroparesis exhibited reduced numbers of contractions in the third (P = 0.05) and fourth (P = 0.0006) quartiles. Diabetics with gastroparesis also showed lower numbers of contractions in the fourth quartile than diabetics with normal emptying (P = 0.02). Diabetics with normal emptying exhibited numbers of contractions that were no different from those of the healthy volunteers in any quartile of colon transit (all P = NS). Numbers of contractions in the final 15 minutes were reduced in diabetics with gastroparesis compared to healthy subjects (P = 0.0005) and diabetics with normal gastric emptying (P = 0.005). Numbers of contractions in the final 15 minutes were no different in diabetics without gastroparesis and healthy volunteers (P = NS).

Numbers of contractions for each colon transit quartile were compared to determine if colon motor defects in diabetic patients with gastroparesis are localized to specific transit phases. Numbers of contractions rose from the first to fourth colon transit quartile in healthy subjects (P<0.0001) and diabetics with normal gastric emptying (P = 0.04)(Fig 5A). Conversely, diabetics with gastroparesis did not exhibit increased contractions from the first to fourth quartiles (P = NS) and showed reduced numbers of contractions in the third (P = 0.05) and fourth (P = 0.0006) quartiles of colon transit vs. the healthy controls. Numbers of contractions were not different in diabetics with normal gastric emptying in any transit quartile versus healthy subjects (all P = NS), but were higher in the fourth transit quartile versus diabetics with gastroparesis (P = 0.02).

Numbers of contractions also were quantified for the final 15 minutes of colon transit prior to anal WMC expulsion. Compared to the first quartile, numbers of contractions were greater in the final 15 minutes in healthy subjects (P<0.0001) and diabetics with normal gastric emptying (P = 0.02) but were not higher in diabetics with gastroparesis (P = NS). Numbers of contractions in the final 15 minutes of colon transit were reduced in diabetics with gastroparesis versus healthy subjects (P = 0.0005) and diabetic patients with normal gastric emptying (P = 0.005)(Fig 5B). Numbers of contractions during this final transit phase were not different in diabetics with normal gastric emptying versus healthy volunteers (P = NS).

### Findings of Multivariate Analyses

Multivariate analyses were performed to identify demographic or clinical variables that might explain differences in numbers of contractions across the subject groups. Numbers of contractions in the healthy controls and two diabetic groups were not related to age (P = 0.67) or sex (P = 0.99) but showed a relation to BMI (P = 0.007). However when age, sex, and BMI were included, differences in numbers of contractions between groups (P = 0.02) and as a function of quartile of transit (P<0.0001) remained significant. Between group differences in the final 15 minutes of transit prior to WMC expulsion remained significant for numbers of contractions (P = 0.002) after adjusting for age, sex, and BMI.

Multivariate analyses also were performed to identify relations of clinical variables specific to the two diabetic subject groups to numbers of colon contractions. Numbers of contractions in the diabetic groups did not relate to fasting glucose (P = 0.65) or use of insulin (P = 0.18), oral hypoglycemics (P = 0.98), or antidepressants (P = 0.54). Diabetic group differences in numbers of contractions in the final 15 minutes of colon transit prior to WMC expulsion remained robust after adjusting for fasting glucose (P = 0.04) and use of insulin (P = 0.02), oral hypoglycemics (P = 0.02), and antidepressants (P = 0.02).

## Discussion

Extensive research has been conducted on gastric emptying impairments in diabetes, but little work has characterized colonic dysfunction with this disorder. Investigations report prevalence rates for constipation in diabetes ranging from 15–60% [5, 6, 8]. In some but not all large population studies, diabetes is a common comorbidity with chronic constipation [7, 9, 10, 29]. Using radiopaque marker or scintigraphy methods, delays in colon transit have been identified in diabetics [11–15]. The introduction of WMC testing has permitted accurate quantification of both transit and contraction numbers in both the stomach and colon [26]. Gastric emptying delays are definable in substantial subsets of those with diabetes of >10 years duration [2–4]. WMC emptying delays correlate well with scintigraphic measures (r = 0.73)[16]. In one analysis, WMC colon transit averaged longer in diabetics with gastroparesis than healthy controls [20]. However, no reports had compared colon transit in diabetics with delayed versus normal gastric emptying and none have enumerated numbers of colon contractions in relation to gastric retention to characterize an association of gastric and colonic dysmotility in diabetes.

Analyses performed for this study on WMC recordings from a parent study of gastric emptying abnormalities in gastroparesis provided novel information relating colon transit delays to gastric emptying among diabetic patients. Colon transit was slowed in diabetics with gastroparesis, while those with normal gastric emptying had colon transit similar to controls. This finding raises the possibility that common factors promote gastric and colon propulsion impairments in diabetes. The lack of correlation of colon transit with fasting glucose levels suggests transit delays are a consequence of fixed motor defects and do not result from acute fluctuations in glycemic control.

Contractile data from this investigation offer insights into motor abnormalities underlying colon transit delays associated with diabetic gastroparesis. These findings are distinct from and complement a prior comparison of colon transit in diabetics with values from healthy controls [20]. Gastroparetics exhibited reductions in overall numbers of colon contractions which correlated with degrees of gastric emptying delays. This finding contrasts with a report which noted no relation of liquid gastric emptying to colonic motility in diabetes [14]. We divided the period of colon transit into quartiles to assess if motor impairments are generalized or selective for the early vs. late phases of WMC transit. Blunting of contractions was noted in the latter colon transit phases only among gastroparetics—especially in the 4^th^ transit quartile and final 15 minutes prior to anal WMC expulsion—raising the possibility that distal regions are more susceptible to damaging effects of diabetes. On multivariate analyses, BMI inversely related to contractility but contractile differences between groups remained significant when this factor was included suggesting colon motor defect in diabetic gastroparesis are not due to obesity. Additionally, transit and contractility were not influenced by hyperglycemia on the morning of testing.

There is precedent for regional differences in damaging effects of diabetes on gut motor function. Gastric emptying is more profoundly impaired than small bowel transit in symptomatic diabetics, but investigations on colon transit have noted inconsistent findings [1]. In some older radioopaque marker studies, delayed left and rectosigmoid colon transit was observed in diabetic patients with preservation of more proximal propulsion [30, 31, 32]. However, other publications report uniform delays in all colon regions in diabetes [4, 11, 15, 33, 34, 35]. It can be postulated that higher numbers of contractions in the distal colon might facilitate evacuation of firmer stools. Our analyses suggest selective deficits in distal colon contractions in diabetics with gastroparesis might contribute to impaired defecation with consequent retardation of transit.

Our observations highlight distinctions in colon contractility between diabetics and individuals with idiopathic constipation which emphasize that disparate motor mechanisms may underlie colon transit impairments in different clinical settings. In a prior study, we noted increased not decreased colon contractions in non-diabetic constipated patients with colon transit values similar to diabetics in this investigation suggesting differences in pathogenesis [23]. In contrast to diabetics, it is possible the exaggerated activity in idiopathic constipation is segmenting in nature and retards rather than propels luminal contents. In recent high resolution manometry studies performed in chronic constipation patients, increases in distal contractility were shown to be retrograde in direction likely causing transit impairments [36].

Clinical and histopathologic studies suggest potential mechanisms for colon motor deficits in diabetic gastroparesis. It is well established that nutrient intake in gastroparesis patients is significantly impaired [37]. Furthermore, manometry-measured gastrocolonic responses to meal ingestion are blunted with diabetes [14]. Taken together, these prior observations provide support for reduced stimulated colonic motility in diabetics with gastroparesis. Colectomy specimens from diabetics with severe constipation exhibit reduced myenteric ganglion size with decreased nNOS, neuropeptide Y, and choline acetyltransferase staining, increased apoptosis, loss of Kit positive cells, and reduced glutathione levels reflective of increased oxidative stress [38, 39, 40]. Colon tissues from diabetic rats show impaired spontaneous and carbamylcholine-evoked contractility with evidence of smooth muscle and neuronal damage, decreased acetylcholine release, altered calcium signaling, and deficiencies of endogenous insulin-like growth factor 1 [41–44].

These comprehensive analyses had limitations. As the WMC has a single pressure sensor, it cannot distinguish mixing from propulsive activity. Thus, conclusions regarding segmentation versus peristalsis are speculative. Secondly, colon transit was parsed into quartiles by time to estimate regional activity but WMC location cannot be verified except just after ileocecal passage and before anal expulsion. Preliminary studies report distinct pH and motor patterns in the proximal and distal colon, but overlaps preclude definite WMC positioning [45]. Thus, our results showing temporal contractility differences cannot be equated with dysfunction in specific colon regions. Nonetheless, contractility impairments in the 4^th^ quartile and final 15 minutes of transit in diabetics with gastroparesis probably can be localized to distal colon.

Questions also can be raised about study design issues. Firstly, the parent study did not query colonic symptoms; it is unknown if diabetics experienced mild constipation (those with <3 bowel movements/week were excluded). Mean colon transit (52 hours) in diabetics with gastroparesis did not satisfy criteria for slow transit constipation (>59 hours), but 5 (42%) exceeded this cutoff and 9 patients (75%) were above the 75^th^ percentile for normal colon transit of 37 hours [19]. It is possible diabetics with colon inertia might show worse contractile deficits than in this study. Secondly, it is conceivable subject characteristics may have influenced some of the findings of this investigation. The sample size was limited by the small cohort from the parent study [16]. The sample sizes were further reduced due to dropout of pressure data during the period of colon transit [20]. Future analyses of larger patient cohorts should be performed with emphasis on correct signal acquisition protocols. The diabetics in this report were older and showed female predominance, while the healthy controls showed a higher percentage of males. However, it is unlikely that the age differences explain the colon contractile impairments in the gastroparesis patients as they were actually younger than the diabetics with normal gastric emptying. Furthermore, it is probable that the female predominance in the diabetics did not explain the contractile differences as diabetic patients with normal gastric emptying showed no contractility differences compared to healthy controls; both of these groups exhibited similar female predominance. Additionally, our prior study in idiopathic constipation showed increased contractility mostly in the latter transit phases in women who were older than controls in that investigation [23]. Thirdly, pre-test glucose values were measured but glycemia was not followed during the period of colon transit and A1c levels were not recorded (although values >10% precluded inclusion). Regardless as values were similar in diabetics with gastroparesis versus normal emptying, it is likely glycemic control had little impact on contractility recorded 1–5 days after WMC ingestion. Finally although all subjects consumed the same initial test meal, diets after discharge from the study centers and medication use were not standardized. It is conceivable that the gastroparesis patients may have consumed less fiber which could have influenced our transit and contractility data [37]. Most drugs that delay transit were stopped before study, but 3 individuals were on tricyclic agents with potential inhibitory properties that could not be stopped. Multivariate analyses showed no impact of antidepressant use on contractile findings.

Our findings have implications for further research into the pathogenesis and possible therapy of colon dysfunction in diabetes. Correlating WMC contractile deficits with colon manometry findings could confirm that propulsive patterns such as high amplitude propagating contractions are impaired in diabetic constipation. In addition to prior studies reporting blunted gastrocolonic responses in diabetic constipation, preliminary WMC analyses have shown reduced gastrocolonic activity in idiopathic constipation [14, 46]. Further WMC studies could determine if improved glycemic control or selected therapies of constipation could enhance gastrocolonic responses to test if such deficits are reversible. Laxatives are the mainstay for treating diabetic constipation. Our demonstration that distal colon contractility is blunted in diabetes suggests that prokinetic agents might be more effective in diabetics than in irritable bowel patients who exhibit increased contractility. The motor stimulant mosapride shows efficacy in increasing stool frequency in diabetics with constipation [47]. More recently, the cholinesterase inhibitor pyridostigmine was reported to accelerate colon transit and improve bowel function in diabetics with constipation many of whom also exhibited autonomic dysfunction [48]. Finally, this investigation highlights that diabetics with gastroparesis commonly have generalized gut transit and motor dysfunction. The implication of this finding is that therapies not targeting the stomach may be beneficial for some gastroparetics with associated colon involvement.

## Conclusions

In conclusion, diabetics with gastroparesis exhibit delayed colon transit with reduced numbers of colon contractions that correlate with delays in gastric emptying. In contrast, diabetics with normal gastric emptying show no impairments of colon function. Contractions increase from early to late colon transit phases in controls and diabetics with normal but not delayed gastric emptying. Reduced contractility in gastroparesis is most prominent in latter transit phases, suggesting selective impairment of distal colon function. These findings suggest the presence of generalized gut dysfunction in some diabetics with gastroparesis, and provide a foundation for future pathophysiologic and therapeutic investigations of bowel disturbances in diabetic patients with associated gastroparesis.

## Supporting Information

S1 File Supporting Information—Protocol Details. The supplemental information file provides details on the protocol from the parent investigation.
(DOCX)

S2 File Supporting Information—Raw Data. This supplemental file provides the raw data acquired for this investigation.
(PDF)

S3 File Supporting Information—Statistical Analyses. This supplemental file includes the statistical analyses performed on the raw data acquired for this investigation.
(PDF)
