# Supplementary material for: Blunting of Colon Contractions in Diabetics with Gastroparesis Quantified by Wireless Motility Capsule Methods
Source: PLoS One. 2015 Oct 28;10(10):e0141183. doi: 10.1371/journal.pone.0141183 (PMC4624915; doi:10.1371/journal.pone.0141183)
Supplement: S1 File — The supplemental information file provides details on the protocol from the parent investigation. (DOCX) [file pone.0141183.s001.docx]

**S1 File. Supplemental Information**

**Protocol Synopsis from Parent Investigation**

After overnight fasting, subjects swallowed a pH-, pressure-, and temperature-sensing WMC (SmartPill Corporation, Buffalo, NY) with 50 ml of water. The capsule measures 26.8 mm x 11.7 mm and transmits at a radio carrier frequency of 434 MHz to a receiver worn by the subject. After WMC ingestion, a low-fat meal (120 g EggBeaters, 2 slices of bread, 30 grams of strawberry jam, and 120 ml of water; 255 kcal, 72% carbohydrate, 24% protein, 2% fat, and 2% fiber) radiolabeled with 1 mCi ^99m^Tc-sulfur colloid was consumed. Data from scintigraphic measures of gastric emptying were not included in the analyses for this investigation. Subjects remained awake during the first eight hours to prevent transit inhibition by sleep. Six hours after WMC ingestion, a 237 ml liquid meal (Ensure, Abbott Laboratories, Abbott Park, IL) was consumed. Subjects were discharged eight hours after WMC ingestion and wore the receiver for up to 13 days or until capsule expulsion was visualized. A formal radiography protocol was followed to confirm capsule passage if needed.

Luminal pH was measured every 5 seconds for the first 24 hours, every 10 seconds from 24-48 hours, and every 2.5 minutes after 48 hours; pH changes from 0.05-9.0 were detected with a sensitivity of +0.5 pH units. Pressure measurements from 0-350 mmHg were acquired every 0.5 seconds during the first 24 hours and every 1 second thereafter; pressure determinations were accurate to +5 mmHg for values <100 mmHg and +10% for pressures >100 mmHg. Temperatures from 25-49^o^C were obtained every 20 seconds in the first 24 hours and every 40 seconds thereafter and were accurate to +1^o^C.
