# Supplementary material for: Blunting of Colon Contractions in Diabetics with Gastroparesis Quantified by Wireless Motility Capsule Methods
Source: PLoS One. 2015 Oct 28;10(10):e0141183. doi: 10.1371/journal.pone.0141183 (PMC4624915; doi:10.1371/journal.pone.0141183)
Supplement: S2 File — This supplemental file provides the raw data acquired for this investigation. (PDF) [file pone.0141183.s002.pdf]

															>25 mmHg					
ID	Group	Sex	Age	BMI	Glucose	Insulin	Oral hypo	Antidep	TCAtetra	NERI	Opiate	GRT	Capped Gl	CTT	Q1num	Q2num	Q3num	Q4num	Lastnum	Allnum
1005	HV	m	55	24								195	195	71	3.38	5.92	1.71	17.75	20	28.76
1009	HV	f	30	20								-5	-5	2197	2.21	14.58	9.75	12.18	4	38.72
1014	HV	m	25	23								218	218	673	12.12	0.8	5.53	15.69	27	34.14
1015	HV	f	22	22								256	256	1655	3.26	0.91	5.29	14.9	16	24.36
1016	HV	f	21	22								301	301	2417	8.04	14.75	21.99	8.66	10	53.44
1017	HV	m	20	24								122	122	308	3.12	7.79	7.79	11.3	8	30
1018	HV	m	27	22								208	208	207	0	2.32	4.06	13.62	15	20
1020	HV	m	34	29								199	199	1273	0.42	3.82	9.62	4.34	12	18.2
1022	HV	f	23	27								211	211	2593	1.57	4.88	10.44	2.59	15	19.48
2006	HV	m	26	22								279	279	949	1.01	0.32	0.44	6.89	25	8.66
2007	HV	f	46	28								229	229	1145	10.27	0.89	8.7	6.92	9	26.78
2011	HV	m	38	27								304	304	766	2.58	1.17	3.99	8.15	17	15.89
2012	HV	f	49	28								145	145	137	10.95	7.88	2.63	5.69	4	27.15
2015	HV	f	22	23								213	213	2338	11.78	5.9	6.42	5.93	28	30.03
2016	HV	f	34	22								175	175	1482	4.9	1.21	2.02	9.19	14	17.32
2018	HV	m	20	23								195	195	813	6.57	0.3	0	2.14	25	9.01
3003	HV	m	22	29								166	166	1438	32.75	55.62	2.84	11.02	11	102.23
3004	HV	m	22	29								1	1	638	5.17	8.84	18.9	23.79	31	56.7
3005	HV	m	23	26								210	210	1627	6.38	1.99	2.29	11.58	41	22.24
3006	HV	f	47	24								161	161	4170	2.23	4.85	4.68	6.16	3	17.92
3007	HV	m	20	30								210	210	1614	7.58	10.78	23.61	26.25	47	68.22
3008	HV	m	47	24								235	235	2172	0.52	3.04	7.1	3.4	14	14.06
3011	HV	f	22	18								167	167	1290	0.84	1.49	0.47	5.4	2	8.2
3012	HV	m	23	27								200	200	1740	6.52	1	2.62	9	65	19.14
3013	HV	m	28	32								165	165	981	26.24	32.78	9.17	20.24	5	88.43
3015	HV	f	49	23								227	227	2541	3.07	5.81	8.81	8.6	27	26.29
4002	HV	m	26	32								234	234	845	6.67	2.98	2.91	4.19	9	16.75
4009	HV	m	32	28								204	204	150	3.6	3.6	8	14	18	29.2
4013	HV	m	27	24								221	221	1513	0.12	4.8	14.67	7.3	2	26.89
4014	HV	m	21	23								227	227	764	4.4	2.75	7.3	1.49	5	15.94
6001	HV	m	53	28								219	219	1399	5.15	6.86	10.98	7.25	36	30.24
6002	HV	m	44	41								94	94	205	7.32	35.54	20.78	12	5	75.64
6003	HV	m	25	25								225	225	135	1.33	1.33	2.67	19.56	27	24.89
7001	HV	m	27	24								269	269	1577	1.9	1.37	7.31	4.03	5	14.61
7006	HV	f	22	23								983	360	237	4.56	4.05	8.35	23.54	5	40.5
7007	HV	m	42	33								1	1	425	3.53	5.36	6.21	13.41	9	28.51
7009	HV	f	27	22								226	226	2325	1.06	7.23	2.43	9.96	14	20.68
7010	HV	m	19	27								255	255	3233	1.56	1.08	4.88	6.31	0	13.83
7019	HV	m	54	26								215	215	860	4.26	12.84	1.67	12.77	44	31.54
7023	HV	f	54	29								265	265	578	8.72	1.45	5.71	15.78	49	31.66
7029	HV	f	41	31								198	198	2531	2.09	6.59	3.89	8.51	12	21.08
1026	DM-Norma	m	53	28		n	n	n			n	262	262	1336	4.13	1.21	8.76	10.56	35	24.66
7017	DM-Norma	f	66	29	75	y	n	y	y	y	n	193	193	590	6.92	4.27	4.27	17.08	3	32.54
7030	DM-Norma	f	50	24	82	y	n	n			n	200	200	2745	6.34	11.43	6.64	15.54	38	39.95
7022	DM-Norma	f	37	27	124	n	n	n			n	249	249	3000	0.76	1.5	0.9	1.4	3	4.56
7018	DM-Norma	f	53	28	155	y	n	n			n	259	259	1226	2.69	1.22	7.19	10.86	11	21.96
7008	DM-Norma	f	57	42	128	n	y	n			n	23	23	4020	1.7	9.03	6.21	6.4	7	23.34
2025	DM-Norma	f	56	22	150	n	y	n			n	217	217	883	4.76	0.75	1.56	6.73	28	13.8
7027	DM-Norma	f	59	30	168	y	n	y	y	y	n	-1	-1	1213	16.03	13.75	2.97	9.94	44	42.69
7026	DM-Delaye	f	41	32	215	y	y	n			n	320	320	5262	5.42	3.71	6.65	7.66	14	23.44
2027	DM-Delaye	f	39	27	95	y	n	n			n	339	339	3628	3.82	2.75	5.09	12.25	13	23.91
7011	DM-Delaye	m	36	30	78	n	n	n			n	1304	360	1909	2.89	6.51	23.76	11.03	3	44.19
1024	DM-Delaye	f	28	36	112	y	y	n			n	1638	360	3840	2.86	5.7	0.38	5.64	3	14.58
7002	DM-Delaye	f	34	30	215	y	n	n			n	10376	360	976	2.46	0.74	0.25	0.68	0	4.13
7004	DM-Delaye	f	62	26	41	y	n	y	y	y	n	10363	360	4256	0.52	0.72	4.44	4.69	4	10.37
7031	DM-Delaye	m	42	27	175	n	n	n			n	2499	360	7166	3.64	5.58	2.7	3.67	10	15.59
7033	DM-Delaye	f	37	18	83	n	n	y	n	n	n	2666	360	2261	1.03	0.77	0.61	1.7	0	4.11
7024	DM-Delaye	m	48	28	192	y	n	n			n	1691	360	3091	0.7	4.46	1.57	2	2	8.73
7003	DM-Delaye	f	54	22	115	y	n	n			n	1041	360	3272	1.16	2.81	3.7	3.91	14	11.58
7005	DM-Delaye	m	52	24	110	y	n	y	n	y	n	1115	360	1382	7.47	10.16	3.13	2.39	4	23.15
7020	DM-Delaye	f	26	34	140	y	n	y	n	y	y	1275	360	542	4.21	11.29	2.44	3.21	0	21.15
