# Supplementary material for: Blunting of Colon Contractions in Diabetics with Gastroparesis Quantified by Wireless Motility Capsule Methods
Source: PLoS One. 2015 Oct 28;10(10):e0141183. doi: 10.1371/journal.pone.0141183 (PMC4624915; doi:10.1371/journal.pone.0141183)
Supplement: S3 File — This supplemental file includes the statistical analyses performed on the raw data acquired for this investigation. (PDF) [file pone.0141183.s003.pdf]

2. Raw data

Raw data

Obs	ID	Group	Sex	Age	BMI	Lastnum	LastAUC
1	1005	HV	m	55	24	20	2594.30
2	1009	HV	f	30	20	4	911.60
3	1014	HV	m	25	23	27	6076.20
4	1015	HV	f	22	22	16	4251.50
5	1016	HV	f	21	22	10	4019.20
6	1017	HV	m	20	24	8	1607.40
7	1018	HV	m	27	22	15	5783.20
8	1020	HV	m	34	29	12	3604.70
9	1022	HV	f	23	27	15	1286.80
10	2006	HV	m	26	22	25	9742.70
11	2007	HV	f	46	28	9	2580.00
12	2011	HV	m	38	27	17	4555.10
13	2012	HV	f	49	28	4	299.20
14	2015	HV	f	22	23	28	8730.80
15	2016	HV	f	34	22	14	3998.00
16	2018	HV	m	20	23	25	1929.60
17	3003	HV	m	22	29	11	1442.60
18	3004	HV	m	22	29	31	1409.97
19	3005	HV	m	23	26	41	8308.30
20	3006	HV	f	47	24	3	357.30
21	3007	HV	m	20	30	47	16731.60
22	3008	HV	m	47	24	14	3164.50
23	3011	HV	f	22	18	2	276.10
24	3012	HV	m	23	27	65	6139.60
25	3013	HV	m	28	32	5	617.10
26	3015	HV	f	49	23	27	8266.10
27	4002	HV	m	26	32	9	17966.60
28	4009	HV	m	32	28	18	2813.00
29	4013	HV	m	27	24	2	344.30
30	4014	HV	m	21	23	5	300.30

Raw data

Obs	ID	Group	Sex	Age	BMI	Lastnum	LastAUC
31	6001	HV	m	53	28	36	6356.90
32	6002	HV	m	44	41	5	370.40
33	6003	HV	m	25	25	27	4402.20
34	7001	HV	m	27	24	5	811.20
35	7006	HV	f	22	23	5	1496.00
36	7007	HV	m	42	33	9	485.10
37	7009	HV	f	27	22	14	3567.10
38	7010	HV	m	19	27	0	0.00
39	7019	HV	m	54	26	44	8237.90
40	7023	HV	f	54	29	49	4075.40
41	7029	HV	f	41	31	12	2323.20
42	1026	DM-Normal	m	53	28	35	7357.90
43	7003	DM-Normal	f	66	29	3	190.00
44	7005	DM-Normal	f	50	24	38	13247.70
45	7017	DM-Normal	f	37	27	3	234.30
46	7026	DM-Normal	f	53	28	11	4182.10
47	7030	DM-Normal	f	57	42	7	2069.20
48	7022	DM-Normal	f	56	22	28	6705.50
49	7020	DM-Normal	f	59	30	44	13655.40
50	7018	DM-Delayed	f	41	32	14	3577.70
51	7008	DM-Delayed	f	39	27	13	7120.80
52	2025	DM-Delayed	m	36	30	3	796.50
53	7027	DM-Delayed	f	28	36	3	922.20
54	7011	DM-Delayed	f	34	30	0	0.00
55	1024	DM-Delayed	f	62	26	4	1229.70
56	2027	DM-Delayed	m	42	27	10	4010.70
57	7002	DM-Delayed	f	37	18	0	0.00
58	7004	DM-Delayed	m	48	28	2	404.10
59	7031	DM-Delayed	f	54	22	14	9808.70

Raw data

Obs	ID	Group	Sex	Age	BMI	Lastnum	LastAUC
60	7033	DM-Delayed	m	52	24	4	5334.20
61	7024	DM-Delayed	f	26	34	0	0.00

Raw data

Obs	ID	Group	Sex	Age	BMI	time	Qnum	Qauc
1	1005	HV	m	55	24	1st quartile	3.38	323.5
2	1005	HV	m	55	24	2nd quartile	5.92	2862.4
3	1005	HV	m	55	24	3rd quartile	1.71	240.3
4	1005	HV	m	55	24	4th quartile	17.75	2816.1
5	1009	HV	f	30	20	1st quartile	2.21	434.5
6	1009	HV	f	30	20	2nd quartile	14.58	4027.3
7	1009	HV	f	30	20	3rd quartile	9.75	2282.3
8	1009	HV	f	30	20	4th quartile	12.18	3542.8
9	1014	HV	m	25	23	1st quartile	12.12	1723.7
10	1014	HV	m	25	23	2nd quartile	0.80	258.7
11	1014	HV	m	25	23	3rd quartile	5.53	1770.5
12	1014	HV	m	25	23	4th quartile	15.69	3321.5
13	1015	HV	f	22	22	1st quartile	3.26	257.8
14	1015	HV	f	22	22	2nd quartile	0.91	122.1
15	1015	HV	f	22	22	3rd quartile	5.29	1727.6
16	1015	HV	f	22	22	4th quartile	14.90	5183.7
17	1016	HV	f	21	22	1st quartile	8.04	832.0
18	1016	HV	f	21	22	2nd quartile	14.75	3719.0
19	1016	HV	f	21	22	3rd quartile	21.99	5091.3
20	1016	HV	f	21	22	4th quartile	8.66	1678.4
21	1017	HV	m	20	24	1st quartile	3.12	281.9
22	1017	HV	m	20	24	2nd quartile	7.79	630.3
23	1017	HV	m	20	24	3rd quartile	7.79	1029.5
24	1017	HV	m	20	24	4th quartile	11.30	6566.8
25	1018	HV	m	27	22	1st quartile	0.00	0.0
26	1018	HV	m	27	22	2nd quartile	2.32	145.8
27	1018	HV	m	27	22	3rd quartile	4.06	520.1
28	1018	HV	m	27	22	4th quartile	13.62	3896.0
29	1020	HV	m	34	29	1st quartile	0.42	51.3
30	1020	HV	m	34	29	2nd quartile	3.82	314.0

Raw data

Obs	ID	Group	Sex	Age	BMI	time	Qnum	Qauc
31	1020	HV	m	34	29	3rd quartile	9.62	1514.7
32	1020	HV	m	34	29	4th quartile	4.34	856.0
33	1022	HV	f	23	27	1st quartile	1.57	117.2
34	1022	HV	f	23	27	2nd quartile	4.88	1196.8
35	1022	HV	f	23	27	3rd quartile	10.44	2620.4
36	1022	HV	f	23	27	4th quartile	2.59	635.9
37	2006	HV	m	26	22	1st quartile	1.01	139.6
38	2006	HV	m	26	22	2nd quartile	0.32	429.4
39	2006	HV	m	26	22	3rd quartile	0.44	43.6
40	2006	HV	m	26	22	4th quartile	6.89	2285.1
41	2007	HV	f	46	28	1st quartile	10.27	710.9
42	2007	HV	f	46	28	2nd quartile	0.89	128.3
43	2007	HV	f	46	28	3rd quartile	8.70	1583.8
44	2007	HV	f	46	28	4th quartile	6.92	1512.8
45	2011	HV	m	38	27	1st quartile	2.58	234.0
46	2011	HV	m	38	27	2nd quartile	1.17	188.9
47	2011	HV	m	38	27	3rd quartile	3.99	480.9
48	2011	HV	m	38	27	4th quartile	8.15	1659.7
49	2012	HV	f	49	28	1st quartile	10.95	1637.2
50	2012	HV	f	49	28	2nd quartile	7.88	725.5
51	2012	HV	f	49	28	3rd quartile	2.63	149.9
52	2012	HV	f	49	28	4th quartile	5.69	579.5
53	2015	HV	f	22	23	1st quartile	11.78	1112.6
54	2015	HV	f	22	23	2nd quartile	5.90	988.2
55	2015	HV	f	22	23	3rd quartile	6.42	1295.1
56	2015	HV	f	22	23	4th quartile	5.93	1884.2
57	2016	HV	f	34	22	1st quartile	4.90	328.6
58	2016	HV	f	34	22	2nd quartile	1.21	63.0
59	2016	HV	f	34	22	3rd quartile	2.02	154.4
60	2016	HV	f	34	22	4th quartile	9.19	1750.2

Raw data

Obs	ID	Group	Sex	Age	BMI	time	Qnum	Qauc
61	2018	HV	m	20	23	1st quartile	6.57	664.6
62	2018	HV	m	20	23	2nd quartile	0.30	24.7
63	2018	HV	m	20	23	3rd quartile	0.00	0.0
64	2018	HV	m	20	23	4th quartile	2.14	163.9
65	3003	HV	m	22	29	1st quartile	32.75	2564.3
66	3003	HV	m	22	29	2nd quartile	55.62	3727.8
67	3003	HV	m	22	29	3rd quartile	2.84	269.6
68	3003	HV	m	22	29	4th quartile	11.02	1735.9
69	3004	HV	m	22	29	1st quartile	5.17	410.6
70	3004	HV	m	22	29	2nd quartile	8.84	1113.4
71	3004	HV	m	22	29	3rd quartile	18.90	2098.7
72	3004	HV	m	22	29	4th quartile	23.79	1874.8
73	3005	HV	m	23	26	1st quartile	6.38	529.6
74	3005	HV	m	23	26	2nd quartile	1.99	133.2
75	3005	HV	m	23	26	3rd quartile	2.29	861.1
76	3005	HV	m	23	26	4th quartile	11.58	7854.7
77	3006	HV	f	47	24	1st quartile	2.23	370.0
78	3006	HV	f	47	24	2nd quartile	4.85	1074.3
79	3006	HV	f	47	24	3rd quartile	4.68	1123.7
80	3006	HV	f	47	24	4th quartile	6.16	1780.9
81	3007	HV	m	20	30	1st quartile	7.58	637.4
82	3007	HV	m	20	30	2nd quartile	10.78	1848.3
83	3007	HV	m	20	30	3rd quartile	23.61	5232.4
84	3007	HV	m	20	30	4th quartile	26.25	5886.3
85	3008	HV	m	47	24	1st quartile	0.52	25.5
86	3008	HV	m	47	24	2nd quartile	3.04	294.9
87	3008	HV	m	47	24	3rd quartile	7.10	1319.3
88	3008	HV	m	47	24	4th quartile	3.40	923.4
89	3011	HV	f	22	18	1st quartile	0.84	46.5
90	3011	HV	f	22	18	2nd quartile	1.49	127.8

Raw data

Obs	ID	Group	Sex	Age	BMI	time	Qnum	Qauc
91	3011	HV	f	22	18	3rd quartile	0.47	67.5
92	3011	HV	f	22	18	4th quartile	5.40	1467.0
93	3012	HV	m	23	27	1st quartile	6.52	328.2
94	3012	HV	m	23	27	2nd quartile	1.00	46.7
95	3012	HV	m	23	27	3rd quartile	2.62	227.3
96	3012	HV	m	23	27	4th quartile	9.00	1176.1
97	3013	HV	m	28	32	1st quartile	26.24	4537.2
98	3013	HV	m	28	32	2nd quartile	32.78	6724.0
99	3013	HV	m	28	32	3rd quartile	9.17	4118.1
100	3013	HV	m	28	32	4th quartile	20.24	3940.9
101	3015	HV	f	49	23	1st quartile	3.07	300.1
102	3015	HV	f	49	23	2nd quartile	5.81	1268.3
103	3015	HV	f	49	23	3rd quartile	8.81	2221.8
104	3015	HV	f	49	23	4th quartile	8.60	2519.4
105	4002	HV	m	26	32	1st quartile	6.67	2221.5
106	4002	HV	m	26	32	2nd quartile	2.98	964.5
107	4002	HV	m	26	32	3rd quartile	2.91	2947.0
108	4002	HV	m	26	32	4th quartile	4.19	2750.6
109	4009	HV	m	32	28	1st quartile	3.60	480.3
110	4009	HV	m	32	28	2nd quartile	3.60	324.6
111	4009	HV	m	32	28	3rd quartile	8.00	772.2
112	4009	HV	m	32	28	4th quartile	14.00	2014.0
113	4013	HV	m	27	24	1st quartile	0.12	10.1
114	4013	HV	m	27	24	2nd quartile	4.80	1906.8
115	4013	HV	m	27	24	3rd quartile	14.67	7791.7
116	4013	HV	m	27	24	4th quartile	7.30	1865.4
117	4014	HV	m	21	23	1st quartile	4.40	730.8
118	4014	HV	m	21	23	2nd quartile	2.75	693.5
119	4014	HV	m	21	23	3rd quartile	7.30	1665.9
120	4014	HV	m	21	23	4th quartile	1.49	97.1

Raw data

Obs	ID	Group	Sex	Age	BMI	time	Qnum	Qauc
121	6001	HV	m	53	28	1st quartile	5.15	530.5
122	6001	HV	m	53	28	2nd quartile	6.86	1790.2
123	6001	HV	m	53	28	3rd quartile	10.98	1755.5
124	6001	HV	m	53	28	4th quartile	7.25	752.1
125	6002	HV	m	44	41	1st quartile	7.32	1160.0
126	6002	HV	m	44	41	2nd quartile	35.54	3839.3
127	6002	HV	m	44	41	3rd quartile	20.78	2140.1
128	6002	HV	m	44	41	4th quartile	12.00	1184.9
129	6003	HV	m	25	25	1st quartile	1.33	68.4
130	6003	HV	m	25	25	2nd quartile	1.33	41.4
131	6003	HV	m	25	25	3rd quartile	2.67	179.2
132	6003	HV	m	25	25	4th quartile	19.56	3282.8
133	7001	HV	m	27	24	1st quartile	1.90	178.5
134	7001	HV	m	27	24	2nd quartile	1.37	193.4
135	7001	HV	m	27	24	3rd quartile	7.31	2163.6
136	7001	HV	m	27	24	4th quartile	4.03	1140.2
137	7006	HV	f	22	23	1st quartile	4.56	812.4
138	7006	HV	f	22	23	2nd quartile	4.05	1400.0
139	7006	HV	f	22	23	3rd quartile	8.35	2676.1
140	7006	HV	f	22	23	4th quartile	23.54	6948.5
141	7007	HV	m	42	33	1st quartile	3.53	394.3
142	7007	HV	m	42	33	2nd quartile	5.36	674.0
143	7007	HV	m	42	33	3rd quartile	6.21	893.5
144	7007	HV	m	42	33	4th quartile	13.41	1267.3
145	7009	HV	f	27	22	1st quartile	1.06	73.3
146	7009	HV	f	27	22	2nd quartile	7.23	912.1
147	7009	HV	f	27	22	3rd quartile	2.43	406.4
148	7009	HV	f	27	22	4th quartile	9.96	2247.9
149	7010	HV	m	19	27	1st quartile	1.56	85.7
150	7010	HV	m	19	27	2nd quartile	1.08	87.3

Raw data

Obs	ID	Group	Sex	Age	BMI	time	Qnum	Qauc
151	7010	HV	m	19	27	3rd quartile	4.88	1416.5
152	7010	HV	m	19	27	4th quartile	6.31	1538.6
153	7019	HV	m	54	26	1st quartile	4.26	432.6
154	7019	HV	m	54	26	2nd quartile	12.84	1680.3
155	7019	HV	m	54	26	3rd quartile	1.67	273.1
156	7019	HV	m	54	26	4th quartile	12.77	2707.2
157	7023	HV	f	54	29	1st quartile	8.72	415.0
158	7023	HV	f	54	29	2nd quartile	1.45	104.7
159	7023	HV	f	54	29	3rd quartile	5.71	454.7
160	7023	HV	f	54	29	4th quartile	15.78	1159.3
161	7029	HV	f	41	31	1st quartile	2.09	376.4
162	7029	HV	f	41	31	2nd quartile	6.59	1500.8
163	7029	HV	f	41	31	3rd quartile	3.89	981.3
164	7029	HV	f	41	31	4th quartile	8.51	3299.8
165	1026	DM-Normal	m	53	28	1st quartile	4.13	296.5
166	1026	DM-Normal	m	53	28	2nd quartile	1.21	117.4
167	1026	DM-Normal	m	53	28	3rd quartile	8.76	2003.7
168	1026	DM-Normal	m	53	28	4th quartile	10.56	2460.8
169	7003	DM-Normal	f	66	29	1st quartile	6.92	878.6
170	7003	DM-Normal	f	66	29	2nd quartile	4.27	741.2
171	7003	DM-Normal	f	66	29	3rd quartile	4.27	620.2
172	7003	DM-Normal	f	66	29	4th quartile	17.08	3712.9
173	7005	DM-Normal	f	50	24	1st quartile	6.34	1511.5
174	7005	DM-Normal	f	50	24	2nd quartile	11.43	4452.2
175	7005	DM-Normal	f	50	24	3rd quartile	6.64	3955.0
176	7005	DM-Normal	f	50	24	4th quartile	15.54	9308.4
177	7017	DM-Normal	f	37	27	1st quartile	0.76	38.5
178	7017	DM-Normal	f	37	27	2nd quartile	1.50	94.7
179	7017	DM-Normal	f	37	27	3rd quartile	0.90	118.9
180	7017	DM-Normal	f	37	27	4th quartile	1.40	184.3

Raw data

Obs	ID	Group	Sex	Age	BMI	time	Qnum	Qauc
181	7026	DM-Normal	f	53	28	1st quartile	2.69	192.4
182	7026	DM-Normal	f	53	28	2nd quartile	1.22	134.6
183	7026	DM-Normal	f	53	28	3rd quartile	7.19	1126.6
184	7026	DM-Normal	f	53	28	4th quartile	10.86	2733.9
185	7030	DM-Normal	f	57	42	1st quartile	1.70	312.1
186	7030	DM-Normal	f	57	42	2nd quartile	9.03	1237.3
187	7030	DM-Normal	f	57	42	3rd quartile	6.21	2011.8
188	7030	DM-Normal	f	57	42	4th quartile	6.40	1887.5
189	7022	DM-Normal	f	56	22	1st quartile	4.76	579.3
190	7022	DM-Normal	f	56	22	2nd quartile	0.75	79.8
191	7022	DM-Normal	f	56	22	3rd quartile	1.56	159.5
192	7022	DM-Normal	f	56	22	4th quartile	6.73	1271.7
193	7020	DM-Normal	f	59	30	1st quartile	16.03	1842.0
194	7020	DM-Normal	f	59	30	2nd quartile	13.75	4744.6
195	7020	DM-Normal	f	59	30	3rd quartile	2.97	512.0
196	7020	DM-Normal	f	59	30	4th quartile	9.94	2180.1
197	7018	DM-Delayed	f	41	32	1st quartile	5.42	447.9
198	7018	DM-Delayed	f	41	32	2nd quartile	3.71	616.7
199	7018	DM-Delayed	f	41	32	3rd quartile	6.65	1471.5
200	7018	DM-Delayed	f	41	32	4th quartile	7.66	2196.0
201	7008	DM-Delayed	f	39	27	1st quartile	3.82	249.7
202	7008	DM-Delayed	f	39	27	2nd quartile	2.75	265.0
203	7008	DM-Delayed	f	39	27	3rd quartile	5.09	1643.1
204	7008	DM-Delayed	f	39	27	4th quartile	12.25	6012.1
205	2025	DM-Delayed	m	36	30	1st quartile	2.89	682.7
206	2025	DM-Delayed	m	36	30	2nd quartile	6.51	2286.1
207	2025	DM-Delayed	m	36	30	3rd quartile	23.76	12534.1
208	2025	DM-Delayed	m	36	30	4th quartile	11.03	4331.6
209	7027	DM-Delayed	f	28	36	1st quartile	2.86	703.7
210	7027	DM-Delayed	f	28	36	2nd quartile	5.70	1240.5

Raw data

Obs	ID	Group	Sex	Age	BMI	time	Qnum	Qauc
211	7027	DM-Delayed	f	28	36	3rd quartile	0.38	949.7
212	7027	DM-Delayed	f	28	36	4th quartile	5.64	1933.0
213	7011	DM-Delayed	f	34	30	1st quartile	2.46	319.9
214	7011	DM-Delayed	f	34	30	2nd quartile	0.74	2588.2
215	7011	DM-Delayed	f	34	30	3rd quartile	0.25	27.3
216	7011	DM-Delayed	f	34	30	4th quartile	0.68	333.7
217	1024	DM-Delayed	f	62	26	1st quartile	0.52	60.8
218	1024	DM-Delayed	f	62	26	2nd quartile	0.72	146.8
219	1024	DM-Delayed	f	62	26	3rd quartile	4.44	924.3
220	1024	DM-Delayed	f	62	26	4th quartile	4.69	1315.1
221	2027	DM-Delayed	m	42	27	1st quartile	3.64	721.6
222	2027	DM-Delayed	m	42	27	2nd quartile	5.58	1756.1
223	2027	DM-Delayed	m	42	27	3rd quartile	2.70	2328.1
224	2027	DM-Delayed	m	42	27	4th quartile	3.67	3367.3
225	7002	DM-Delayed	f	37	18	1st quartile	1.03	163.7
226	7002	DM-Delayed	f	37	18	2nd quartile	0.77	258.9
227	7002	DM-Delayed	f	37	18	3rd quartile	0.61	1137.6
228	7002	DM-Delayed	f	37	18	4th quartile	1.70	378.8
229	7004	DM-Delayed	m	48	28	1st quartile	0.70	96.3
230	7004	DM-Delayed	m	48	28	2nd quartile	4.46	1366.5
231	7004	DM-Delayed	m	48	28	3rd quartile	1.57	307.2
232	7004	DM-Delayed	m	48	28	4th quartile	2.00	399.4
233	7031	DM-Delayed	f	54	22	1st quartile	1.16	179.2
234	7031	DM-Delayed	f	54	22	2nd quartile	2.81	795.4
235	7031	DM-Delayed	f	54	22	3rd quartile	3.70	1002.8
236	7031	DM-Delayed	f	54	22	4th quartile	3.91	1444.7
237	7033	DM-Delayed	m	52	24	1st quartile	7.47	2167.8
238	7033	DM-Delayed	m	52	24	2nd quartile	10.16	3061.9
239	7033	DM-Delayed	m	52	24	3rd quartile	3.13	959.0
240	7033	DM-Delayed	m	52	24	4th quartile	2.39	726.4

Raw data

Obs	ID	Group	Sex	Age	BMI	time	Qnum	Qauc
241	7024	DM-Delayed	f	26	34	1st quartile	4.21	416.0
242	7024	DM-Delayed	f	26	34	2nd quartile	11.29	1424.6
243	7024	DM-Delayed	f	26	34	3rd quartile	2.44	189.2
244	7024	DM-Delayed	f	26	34	4th quartile	3.21	281.0

3. Analysis of Number of Contr/15 mins

Analysis of endpoint Qnum by GET quartile and group
Descriptive statistics by variable(s) group time

Analysis Variable : Qnum

Group	time	N Obs	N	Mean	Std Dev	Std Error	Range	Minimum	Maximum
DM-Delayed	1st quartile	12	12	3.015	2.076	0.599	6.950	0.520	7.470
	2nd quartile	12	12	4.600	3.484	1.006	10.570	0.720	11.290
	3rd quartile	12	12	4.560	6.358	1.835	23.510	0.250	23.760
	4th quartile	12	12	4.903	3.667	1.059	11.570	0.680	12.250
DM-Normal	1st quartile	8	8	5.416	4.792	1.694	15.270	0.760	16.030
	2nd quartile	8	8	5.395	5.243	1.854	13.000	0.750	13.750
	3rd quartile	8	8	4.813	2.831	1.001	7.860	0.900	8.760
	4th quartile	8	8	9.814	5.060	1.789	15.680	1.400	17.080
HV	1st quartile	41	41	5.604	6.411	1.001	32.750	0.000	32.750
	2nd quartile	41	41	7.255	10.753	1.679	55.320	0.300	55.620
	3rd quartile	41	41	7.040	5.793	0.905	23.610	0.000	23.610
	4th quartile	41	41	10.524	6.121	0.956	24.760	1.490	26.250

Analysis Variable : Qnum

Group	time	N Obs	5th Pctl	10th Pctl	Lower Quartile	Median	Upper Quartile	90th Pctl	95th Pctl
DM-Delayed	1st quartile	12	0.520	0.700	1.095	2.875	4.015	5.420	7.470
	2nd quartile	12	0.720	0.740	1.760	4.085	6.105	10.160	11.290
	3rd quartile	12	0.250	0.380	1.090	2.915	4.765	6.650	23.760
	4th quartile	12	0.680	1.700	2.195	3.790	6.650	11.030	12.250
DM-Normal	1st quartile	8	0.760	0.760	2.195	4.445	6.630	16.030	16.030
	2nd quartile	8	0.750	0.750	1.215	2.885	10.230	13.750	13.750
	3rd quartile	8	0.900	0.900	2.265	5.240	6.915	8.760	8.760
	4th quartile	8	1.400	1.400	6.565	10.250	13.200	17.080	17.080
HV	1st quartile	41	0.420	0.840	1.900	3.600	6.670	10.950	12.120
	2nd quartile	41	0.800	0.910	1.370	4.050	7.230	14.580	32.780
	3rd quartile	41	0.470	1.710	2.670	5.710	8.810	14.670	20.780
	4th quartile	41	2.590	4.030	6.160	9.000	13.620	19.560	23.540

Analysis of endpoint Qnum by GET quartile and group

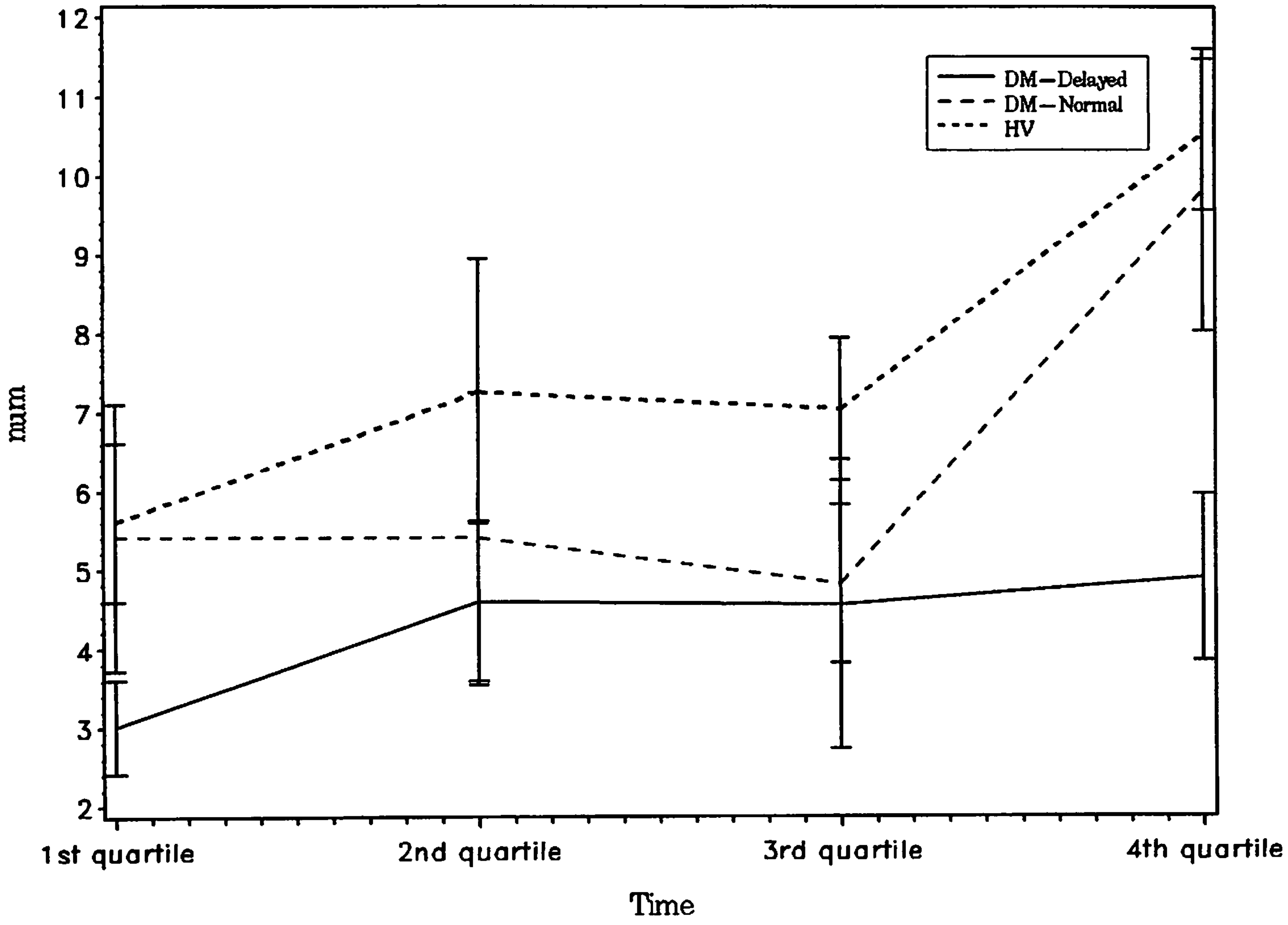

Analysis of endpoint Qnum by GET quartile and group

Overall, within time period, and within group tests based on fitted marginal model

Type 3 Tests of Fixed Effects

Effect	Num DF	Den DF	F Value	Pr > F
Group	2	58	2.85	0.0657
time	3	58	9.32	<.0001
Group*time	6	58	0.84	0.5421

Group	Group	Estimate	P-value
DM-Delayed	DM-Normal	-0.3250	0.1716
DM-Delayed	HV	-0.4033	0.0202
DM-Normal	HV	-0.07825	0.6954

Time	Group	Group	Estimate	P-value
1st quartile	DM-Delayed	DM-Normal	-0.3825	0.2610
1st quartile	DM-Delayed	HV	-0.3092	0.2071
1st quartile	DM-Normal	HV	0.07334	0.7981
2nd quartile	DM-Delayed	DM-Normal	-0.00590	0.9880
2nd quartile	DM-Delayed	HV	-0.1230	0.6642
2nd quartile	DM-Normal	HV	-0.1170	0.7256
3rd quartile	DM-Delayed	DM-Normal	-0.2750	0.4196
3rd quartile	DM-Delayed	HV	-0.4872	0.0498
3rd quartile	DM-Normal	HV	-0.2122	0.4617
4th quartile	DM-Delayed	DM-Normal	-0.6367	0.0204
4th quartile	DM-Delayed	HV	-0.6937	0.0006
4th quartile	DM-Normal	HV	-0.05705	0.8017

Group	Time	Time	Estimate	P-value
DM-Delayed	1st quartile	2nd quartile	-0.2638	0.2965
DM-Delayed	1st quartile	3rd quartile	-0.09063	0.7509
DM-Delayed	1st quartile	4th quartile	-0.3443	0.1390
DM-Delayed	2nd quartile	3rd quartile	0.1732	0.4982
DM-Delayed	2nd quartile	4th quartile	-0.08051	0.7535
DM-Delayed	3rd quartile	4th quartile	-0.2537	0.2413

Analysis of endpoint Qnum by GET quartile and group

Overall, within time period, and within group tests based on fitted marginal model

Group	Time	Time	Estimate	P-value
DM-Normal	1st quartile	2nd quartile	0.1128	0.7143
DM-Normal	1st quartile	3rd quartile	0.01690	0.9614
DM-Normal	1st quartile	4th quartile	-0.5985	0.0375
DM-Normal	2nd quartile	3rd quartile	-0.09592	0.7590
DM-Normal	2nd quartile	4th quartile	-0.7113	0.0266
DM-Normal	3rd quartile	4th quartile	-0.6154	0.0225
HV	1st quartile	2nd quartile	-0.07757	0.5691
HV	1st quartile	3rd quartile	-0.2687	0.0857
HV	1st quartile	4th quartile	-0.7289	<.0001
HV	2nd quartile	3rd quartile	-0.1911	0.1697
HV	2nd quartile	4th quartile	-0.6513	<.0001
HV	3rd quartile	4th quartile	-0.4602	0.0002

Analysis of endpoint Qnum by GET quartile and group
Adjusted for sex and age
Overall, within time period, and within group tests based on fitted marginal model

Type 3 Tests of Fixed Effects

Effect	Num DF	Den DF	F Value	Pr > F
Group	2	56	2.69	0.0770
time	3	56	9.32	<.0001
Group*time	6	56	0.84	0.5423
Sex	1	56	0.27	0.6023
Age	1	56	0.48	0.4917

Group	Group	Estimate	P-value
DM-Delayed	DM-Normal	-0.2909	0.2463
DM-Delayed	HV	-0.4203	0.0253
DM-Normal	HV	-0.1294	0.5988

Time	Group	Group	Estimate	P-value
1st quartile	DM-Delayed	DM-Normal	-0.3484	0.3215
1st quartile	DM-Delayed	HV	-0.3262	0.2037
1st quartile	DM-Normal	HV	0.02218	0.9451
2nd quartile	DM-Delayed	DM-Normal	0.02821	0.9439
2nd quartile	DM-Delayed	HV	-0.1400	0.6310
2nd quartile	DM-Normal	HV	-0.1682	0.6426
3rd quartile	DM-Delayed	DM-Normal	-0.2409	0.4903
3rd quartile	DM-Delayed	HV	-0.5043	0.0508
3rd quartile	DM-Normal	HV	-0.2634	0.4128
4th quartile	DM-Delayed	DM-Normal	-0.6026	0.0350
4th quartile	DM-Delayed	HV	-0.7108	0.0010
4th quartile	DM-Normal	HV	-0.1082	0.6870

Analysis of endpoint Qnum by GET quartile and group
Adjusted for sex and age
Overall, within time period, and within group tests based on fitted marginal model

Group	Time	Time	Estimate	P-value
DM-Delayed	1st quartile	2nd quartile	-0.2638	0.2966
DM-Delayed	1st quartile	3rd quartile	-0.09063	0.7509
DM-Delayed	1st quartile	4th quartile	-0.3443	0.1392
DM-Delayed	2nd quartile	3rd quartile	0.1732	0.4983
DM-Delayed	2nd quartile	4th quartile	-0.08051	0.7536
DM-Delayed	3rd quartile	4th quartile	-0.2537	0.2415
DM-Normal	1st quartile	2nd quartile	0.1128	0.7143
DM-Normal	1st quartile	3rd quartile	0.01690	0.9614
DM-Normal	1st quartile	4th quartile	-0.5985	0.0376
DM-Normal	2nd quartile	3rd quartile	-0.09592	0.7590
DM-Normal	2nd quartile	4th quartile	-0.7113	0.0267
DM-Normal	3rd quartile	4th quartile	-0.6154	0.0226
HV	1st quartile	2nd quartile	-0.07757	0.5692
HV	1st quartile	3rd quartile	-0.2687	0.0859
HV	1st quartile	4th quartile	-0.7289	<.0001
HV	2nd quartile	3rd quartile	-0.1911	0.1699
HV	2nd quartile	4th quartile	-0.6513	<.0001
HV	3rd quartile	4th quartile	-0.4602	0.0002

Analysis of endpoint Qnum by GET quartile and group
Adjusted for sex, age, and bmi
Overall, within time period, and within group tests based on fitted marginal model

Type 3 Tests of Fixed Effects

Effect	Num DF	Den DF	F Value	Pr > F
Group	2	55	4.16	0.0208
time	3	55	9.32	<.0001
Group*time	6	55	0.84	0.5424
Sex	1	55	0.00	0.9936
Age	1	55	0.18	0.6724
BMI	1	55	7.75	0.0073

Group	Group	Estimate	P-value
DM-Delayed	DM-Normal	-0.2621	0.2606
DM-Delayed	HV	-0.4961	0.0056
DM-Normal	HV	-0.2340	0.3163

Time	Group	Group	Estimate	P-value
1st quartile	DM-Delayed	DM-Normal	-0.3196	0.3457
1st quartile	DM-Delayed	HV	-0.4020	0.1079
1st quartile	DM-Normal	HV	-0.08244	0.7916
2nd quartile	DM-Delayed	DM-Normal	0.05700	0.8797
2nd quartile	DM-Delayed	HV	-0.2158	0.4336
2nd quartile	DM-Normal	HV	-0.2728	0.4271
3rd quartile	DM-Delayed	DM-Normal	-0.2121	0.5312
3rd quartile	DM-Delayed	HV	-0.5801	0.0221
3rd quartile	DM-Normal	HV	-0.3680	0.2416
4th quartile	DM-Delayed	DM-Normal	-0.5738	0.0449
4th quartile	DM-Delayed	HV	-0.7866	0.0003
4th quartile	DM-Normal	HV	-0.2128	0.4293

Analysis of endpoint Qnum by GET quartile and group
Adjusted for sex, age, and bmi
Overall, within time period, and within group tests based on fitted marginal model

Group	Time	Time	Estimate	P-value
DM-Delayed	1st quartile	2nd quartile	-0.2638	0.2967
DM-Delayed	1st quartile	3rd quartile	-0.09063	0.7509
DM-Delayed	1st quartile	4th quartile	-0.3443	0.1393
DM-Delayed	2nd quartile	3rd quartile	0.1732	0.4984
DM-Delayed	2nd quartile	4th quartile	-0.08051	0.7536
DM-Delayed	3rd quartile	4th quartile	-0.2537	0.2416
DM-Normal	1st quartile	2nd quartile	0.1128	0.7144
DM-Normal	1st quartile	3rd quartile	0.01690	0.9614
DM-Normal	1st quartile	4th quartile	-0.5985	0.0377
DM-Normal	2nd quartile	3rd quartile	-0.09592	0.7590
DM-Normal	2nd quartile	4th quartile	-0.7113	0.0268
DM-Normal	3rd quartile	4th quartile	-0.6154	0.0227
HV	1st quartile	2nd quartile	-0.07757	0.5692
HV	1st quartile	3rd quartile	-0.2687	0.0860
HV	1st quartile	4th quartile	-0.7289	<.0001
HV	2nd quartile	3rd quartile	-0.1911	0.1700
HV	2nd quartile	4th quartile	-0.6513	<.0001
HV	3rd quartile	4th quartile	-0.4602	0.0002

Analysis of endpoint Qnum by GET quartile and group - Diabetics only
Adjusted for sex
Overall, within time period, and within group tests based on fitted marginal model

Type 3 Tests of Fixed Effects

Effect	Num DF	Den DF	F Value	Pr > F
Group	1	17	2.69	0.1196
time	3	17	9.24	0.0007
Group*time	3	17	1.50	0.2495
Sex	1	17	1.52	0.2346

Group	Group	Estimate	P-value
DM-Delayed	DM-Normal	-0.3932	0.1196

Time	Group	Group	Estimate	P-value
1st quartile	DM-Delayed	DM-Normal	-0.4507	0.1382
2nd quartile	DM-Delayed	DM-Normal	-0.07407	0.8355
3rd quartile	DM-Delayed	DM-Normal	-0.3431	0.3155
4th quartile	DM-Delayed	DM-Normal	-0.7049	0.0311

Group	Time	Time	Estimate	P-value
DM-Delayed	1st quartile	2nd quartile	-0.2638	0.1825
DM-Delayed	1st quartile	3rd quartile	-0.09063	0.7230
DM-Delayed	1st quartile	4th quartile	-0.3443	0.0799
DM-Delayed	2nd quartile	3rd quartile	0.1732	0.5220
DM-Delayed	2nd quartile	4th quartile	-0.08051	0.7401
DM-Delayed	3rd quartile	4th quartile	-0.2537	0.1107
DM-Normal	1st quartile	2nd quartile	0.1128	0.6336
DM-Normal	1st quartile	3rd quartile	0.01690	0.9569
DM-Normal	1st quartile	4th quartile	-0.5985	0.0171
DM-Normal	2nd quartile	3rd quartile	-0.09592	0.7710
DM-Normal	2nd quartile	4th quartile	-0.7113	0.0264
DM-Normal	3rd quartile	4th quartile	-0.6154	0.0039

Analysis of endpoint Qnum by GET quartile and group - Diabetics only
Adjusted for age
Overall, within time period, and within group tests based on fitted marginal model

Type 3 Tests of Fixed Effects

Effect	Num DF	Den DF	F Value	Pr > F
Group	1	17	0.63	0.4375
time	3	17	9.24	0.0007
Group*time	3	17	1.50	0.2495
Age	1	17	0.46	0.5054

Group	Group	Estimate	P-value
DM-Delayed	DM-Normal	-0.2232	0.4375

Time	Group	Group	Estimate	P-value
1st quartile	DM-Delayed	DM-Normal	-0.2807	0.3961
2nd quartile	DM-Delayed	DM-Normal	0.09593	0.8074
3rd quartile	DM-Delayed	DM-Normal	-0.1731	0.6442
4th quartile	DM-Delayed	DM-Normal	-0.5349	0.1131

Group	Time	Time	Estimate	P-value
DM-Delayed	1st quartile	2nd quartile	-0.2638	0.1825
DM-Delayed	1st quartile	3rd quartile	-0.09063	0.7230
DM-Delayed	1st quartile	4th quartile	-0.3443	0.0799
DM-Delayed	2nd quartile	3rd quartile	0.1732	0.5220
DM-Delayed	2nd quartile	4th quartile	-0.08051	0.7401
DM-Delayed	3rd quartile	4th quartile	-0.2537	0.1107
DM-Normal	1st quartile	2nd quartile	0.1128	0.6336
DM-Normal	1st quartile	3rd quartile	0.01690	0.9569
DM-Normal	1st quartile	4th quartile	-0.5985	0.0171
DM-Normal	2nd quartile	3rd quartile	-0.09592	0.7710
DM-Normal	2nd quartile	4th quartile	-0.7113	0.0264
DM-Normal	3rd quartile	4th quartile	-0.6154	0.0039

Analysis of endpoint Qnum by GET quartile and group - Diabetics only
Adjusted for bmi
Overall, within time period, and within group tests based on fitted marginal model

Type 3 Tests of Fixed Effects

Effect	Num DF	Den DF	F Value	Pr > F
Group	1	17	1.71	0.2084
time	3	17	9.24	0.0007
Group*time	3	17	1.50	0.2495
BMI	1	17	1.10	0.3097

Group	Group	Estimate	P-value
DM-Delayed	DM-Normal	-0.3041	0.2084

Time	Group	Group	Estimate	P-value
1st quartile	DM-Delayed	DM-Normal	-0.3615	0.2229
2nd quartile	DM-Delayed	DM-Normal	0.01506	0.9645
3rd quartile	DM-Delayed	DM-Normal	-0.2540	0.4687
4th quartile	DM-Delayed	DM-Normal	-0.6157	0.0476

Group	Time	Time	Estimate	P-value
DM-Delayed	1st quartile	2nd quartile	-0.2638	0.1825
DM-Delayed	1st quartile	3rd quartile	-0.09063	0.7230
DM-Delayed	1st quartile	4th quartile	-0.3443	0.0799
DM-Delayed	2nd quartile	3rd quartile	0.1732	0.5220
DM-Delayed	2nd quartile	4th quartile	-0.08051	0.7401
DM-Delayed	3rd quartile	4th quartile	-0.2537	0.1107
DM-Normal	1st quartile	2nd quartile	0.1128	0.6336
DM-Normal	1st quartile	3rd quartile	0.01690	0.9569
DM-Normal	1st quartile	4th quartile	-0.5985	0.0171
DM-Normal	2nd quartile	3rd quartile	-0.09592	0.7710
DM-Normal	2nd quartile	4th quartile	-0.7113	0.0264
DM-Normal	3rd quartile	4th quartile	-0.6154	0.0039

Analysis of endpoint Qnum by GET quartile and group - Diabetics only
Adjusted for Glucose
Overall, within time period, and within group tests based on fitted marginal model

Type 3 Tests of Fixed Effects

Effect	Num DF	Den DF	F Value	Pr > F
Group	1	16	1.52	0.2356
time	3	16	9.28	0.0009
Group*time	3	16	1.31	0.3069
glucose	1	16	0.21	0.6498

Group	Group	Estimate	P-value
DM-Delayed	DM-Normal	-0.3153	0.2356

Time	Group	Group	Estimate	P-value
1st quartile	DM-Delayed	DM-Normal	-0.3779	0.2422
2nd quartile	DM-Delayed	DM-Normal	-0.1055	0.7791
3rd quartile	DM-Delayed	DM-Normal	-0.1761	0.6261
4th quartile	DM-Delayed	DM-Normal	-0.6015	0.0631

Group	Time	Time	Estimate	P-value
DM-Delayed	1st quartile	2nd quartile	-0.2638	0.1786
DM-Delayed	1st quartile	3rd quartile	-0.09063	0.7260
DM-Delayed	1st quartile	4th quartile	-0.3443	0.0881
DM-Delayed	2nd quartile	3rd quartile	0.1732	0.5017
DM-Delayed	2nd quartile	4th quartile	-0.08051	0.7367
DM-Delayed	3rd quartile	4th quartile	-0.2537	0.1135
DM-Normal	1st quartile	2nd quartile	0.008636	0.9724
DM-Normal	1st quartile	3rd quartile	0.1112	0.7425
DM-Normal	1st quartile	4th quartile	-0.5679	0.0360
DM-Normal	2nd quartile	3rd quartile	0.1026	0.7599
DM-Normal	2nd quartile	4th quartile	-0.5766	0.0797
DM-Normal	3rd quartile	4th quartile	-0.6791	0.0035

Analysis of endpoint Qnum by GET quartile and group - Diabetics only
Adjusted for Insulin
Overall, within time period, and within group tests based on fitted marginal model

Type 3 Tests of Fixed Effects

Effect	Num DF	Den DF	F Value	Pr > F
Group	1	17	2.91	0.1064
time	3	17	9.24	0.0007
Group*time	3	17	1.50	0.2495
Insulin	1	17	2.00	0.1750

Group	Group	Estimate	P-value
DM-Delayed	DM-Normal	-0.4095	0.1064

Time	Group	Group	Estimate	P-value
1st quartile	DM-Delayed	DM-Normal	-0.4669	0.1069
2nd quartile	DM-Delayed	DM-Normal	-0.09034	0.7982
3rd quartile	DM-Delayed	DM-Normal	-0.3594	0.3326
4th quartile	DM-Delayed	DM-Normal	-0.7211	0.0219

Group	Time	Time	Estimate	P-value
DM-Delayed	1st quartile	2nd quartile	-0.2638	0.1825
DM-Delayed	1st quartile	3rd quartile	-0.09063	0.7230
DM-Delayed	1st quartile	4th quartile	-0.3443	0.0799
DM-Delayed	2nd quartile	3rd quartile	0.1732	0.5220
DM-Delayed	2nd quartile	4th quartile	-0.08051	0.7401
DM-Delayed	3rd quartile	4th quartile	-0.2537	0.1107
DM-Normal	1st quartile	2nd quartile	0.1128	0.6336
DM-Normal	1st quartile	3rd quartile	0.01690	0.9569
DM-Normal	1st quartile	4th quartile	-0.5985	0.0171
DM-Normal	2nd quartile	3rd quartile	-0.09592	0.7710
DM-Normal	2nd quartile	4th quartile	-0.7113	0.0264
DM-Normal	3rd quartile	4th quartile	-0.6154	0.0039

Analysis of endpoint Qnum by GET quartile and group - Diabetics only
Adjusted for Oral_hypo
Overall, within time period, and within group tests based on fitted marginal model

Type 3 Tests of Fixed Effects

Effect	Num DF	Den DF	F Value	Pr > F
Group	1	17	1.80	0.1969
time	3	17	9.24	0.0007
Group*time	3	17	1.50	0.2495
Oral_hypo	1	17	0.00	0.9807

Group	Group	Estimate	P-value
DM-Delayed	DM-Normal	-0.3244	0.1969

Time	Group	Group	Estimate	P-value
1st quartile	DM-Delayed	DM-Normal	-0.3819	0.1975
2nd quartile	DM-Delayed	DM-Normal	-0.00531	0.9882
3rd quartile	DM-Delayed	DM-Normal	-0.2744	0.4385
4th quartile	DM-Delayed	DM-Normal	-0.6361	0.0425

Group	Time	Time	Estimate	P-value
DM-Delayed	1st quartile	2nd quartile	-0.2638	0.1825
DM-Delayed	1st quartile	3rd quartile	-0.09063	0.7230
DM-Delayed	1st quartile	4th quartile	-0.3443	0.0799
DM-Delayed	2nd quartile	3rd quartile	0.1732	0.5220
DM-Delayed	2nd quartile	4th quartile	-0.08051	0.7401
DM-Delayed	3rd quartile	4th quartile	-0.2537	0.1107
DM-Normal	1st quartile	2nd quartile	0.1128	0.6336
DM-Normal	1st quartile	3rd quartile	0.01690	0.9569
DM-Normal	1st quartile	4th quartile	-0.5985	0.0171
DM-Normal	2nd quartile	3rd quartile	-0.09592	0.7710
DM-Normal	2nd quartile	4th quartile	-0.7113	0.0264
DM-Normal	3rd quartile	4th quartile	-0.6154	0.0039

Analysis of endpoint Qnum by GET quartile and group - Diabetics only
Adjusted for Antidep
Overall, within time period, and within group tests based on fitted marginal model

Type 3 Tests of Fixed Effects

Effect	Num DF	Den DF	F Value	Pr > F
Group	1	17	1.98	0.1778
time	3	17	9.24	0.0007
Group*time	3	17	1.50	0.2495
Antidep	1	17	0.38	0.5440

Group	Group	Estimate	P-value
DM-Delayed	DM-Normal	-0.3379	0.1778

Time	Group	Group	Estimate	P-value
1st quartile	DM-Delayed	DM-Normal	-0.3954	0.1715
2nd quartile	DM-Delayed	DM-Normal	-0.01883	0.9577
3rd quartile	DM-Delayed	DM-Normal	-0.2879	0.4236
4th quartile	DM-Delayed	DM-Normal	-0.6496	0.0407

Group	Time	Time	Estimate	P-value
DM-Delayed	1st quartile	2nd quartile	-0.2638	0.1825
DM-Delayed	1st quartile	3rd quartile	-0.09063	0.7230
DM-Delayed	1st quartile	4th quartile	-0.3443	0.0799
DM-Delayed	2nd quartile	3rd quartile	0.1732	0.5220
DM-Delayed	2nd quartile	4th quartile	-0.08051	0.7401
DM-Delayed	3rd quartile	4th quartile	-0.2537	0.1107
DM-Normal	1st quartile	2nd quartile	0.1128	0.6336
DM-Normal	1st quartile	3rd quartile	0.01690	0.9569
DM-Normal	1st quartile	4th quartile	-0.5985	0.0171
DM-Normal	2nd quartile	3rd quartile	-0.09592	0.7710
DM-Normal	2nd quartile	4th quartile	-0.7113	0.0264
DM-Normal	3rd quartile	4th quartile	-0.6154	0.0039

Analysis of endpoint Lastnum by group
Descriptive statistics by variable(s) group

Analysis Variable : Lastnum Lastnum

Group	N Obs	N	Mean	Std Dev	Std Error	Range	Minimum	Maximum
DM-Delayed	12	12	5.583	5.567	1.607	14.000	0.000	14.000
DM-Normal	8	8	21.125	16.932	5.986	41.000	3.000	44.000
HV	41	41	17.927	15.042	2.349	65.000	0.000	65.000

Analysis Variable : Lastnum Lastnum

Group	N Obs	5th Pctl	10th Pctl	Lower Quartile	Median	Upper Quartile	90th Pctl	95th Pctl
DM-Delayed	12	0.000	0.000	1.000	3.500	11.500	14.000	14.000
DM-Normal	8	3.000	3.000	5.000	19.500	36.500	44.000	44.000
HV	41	2.000	4.000	5.000	14.000	27.000	41.000	47.000

Analysis of endpoint Lastnum by group

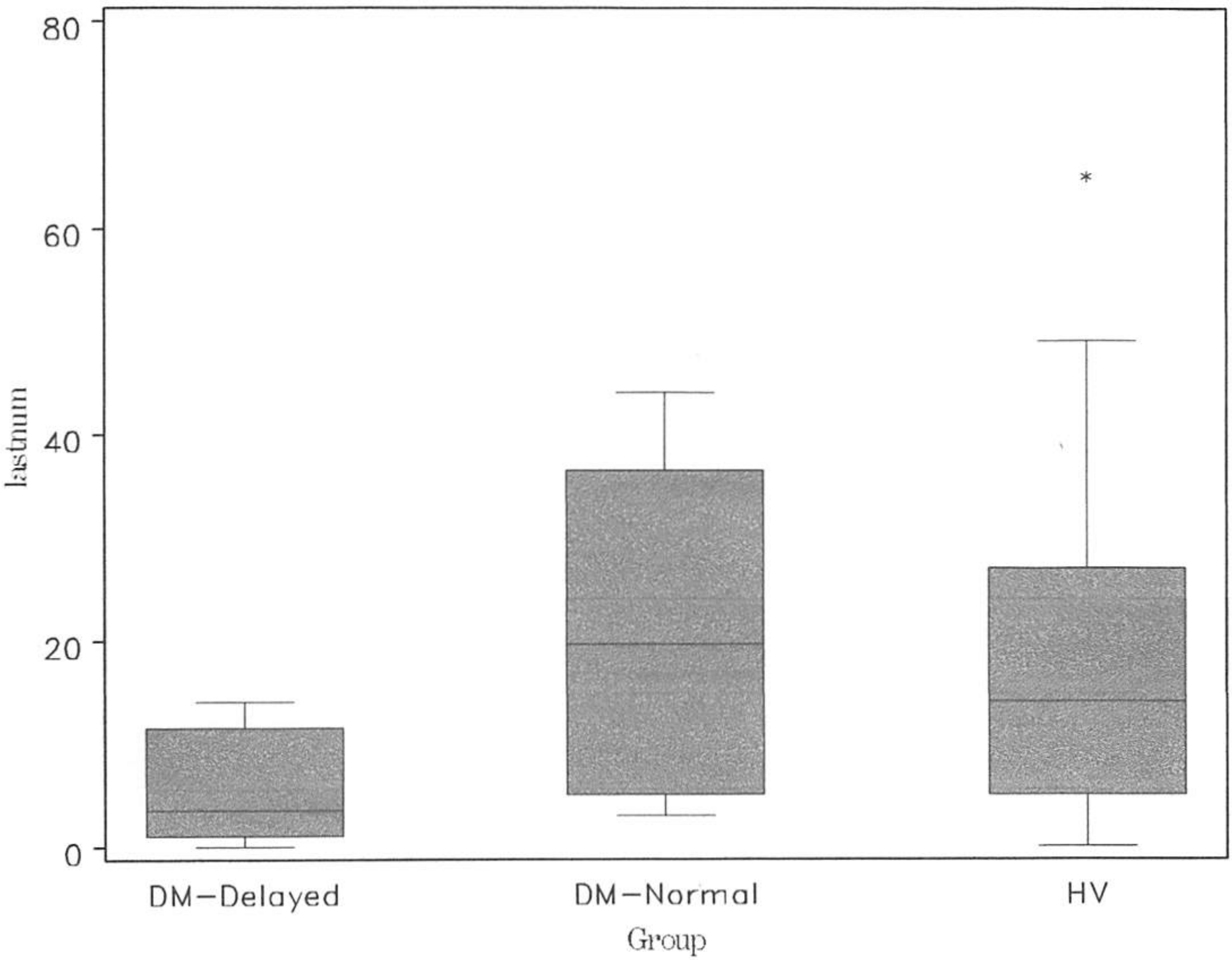

Analysis of endpoint Lastnum by group

Overall and between group tests based on fitted model

Type 3 Tests of Fixed Effects

Effect	Num DF	Den DF	F Value	Pr > F
Group	2	58	7.33	0.0014

Group	Group	Estimate	P-value
DM-Delayed	DM-Normal	-1.2578	0.0050
DM-Delayed	HV	-1.1414	0.0005
DM-Normal	HV	0.1165	0.7507

Analysis of endpoint Lastnum by group
Adjusted for sex and age
Overall and between group tests based on fitted model

Type 3 Tests of Fixed Effects

Effect	Num DF	Den DF	F Value	Pr > F
Group	2	56	7.49	0.0013
Sex	1	56	1.81	0.1842
Age	1	56	2.69	0.1065

Group	Group	Estimate	P-value
DM-Delayed	DM-Normal	-1.1101	0.0157
DM-Delayed	HV	-1.2100	0.0005
DM-Normal	HV	-0.09986	0.8235

Analysis of endpoint Lastnum by group
Adjusted for sex, age, and bmi
Overall and between group tests based on fitted model

Type 3 Tests of Fixed Effects

Effect	Num DF	Den DF	F Value	Pr > F
Group	2	55	7.00	0.0020
Sex	1	55	2.17	0.1464
Age	1	55	2.95	0.0917
BMI	1	55	0.65	0.4223

Group	Group	Estimate	P-value
DM-Delayed	DM-Normal	-1.1264	0.0148
DM-Delayed	HV	-1.1662	0.0010
DM-Normal	HV	-0.03976	0.9304

Analysis of endpoint Lastnum by group - Diabetics only
Adjusted for sex
Overall and between group tests based on fitted model

Type 3 Tests of Fixed Effects

Effect	Num DF	Den DF	F Value	Pr > F
Group	1	17	7.52	0.0139
Sex	1	17	0.59	0.4538

Group	Group	Estimate	P-value
DM-Delayed	DM-Normal	-1.3465	0.0139

Analysis of endpoint Lastnum by group - Diabetics only
Adjusted for age
Overall and between group tests based on fitted model

Type 3 Tests of Fixed Effects				
Effect	Num DF	Den DF	F Value	Pr > F
Group	1	17	2.26	0.1514
Age	1	17	2.30	0.1477

Group	Group	Estimate	P-value
DM-Delayed	DM-Normal	-0.8135	0.1514

Analysis of endpoint Lastnum by group - Diabetics only
Adjusted for bmi
Overall and between group tests based on fitted model

Type 3 Tests of Fixed Effects

Effect	Num DF	Den DF	F Value	Pr > F
Group	1	17	7.22	0.0156
BMI	1	17	0.54	0.4714

Group	Group	Estimate	P-value
DM-Delayed	DM-Normal	-1.2885	0.0156

Analysis of endpoint Lastnum by group - Diabetics only
Adjusted for Glucose
Overall and between group tests based on fitted model

Type 3 Tests of Fixed Effects

Effect	Num DF	Den DF	F Value	Pr > F
Group	1	16	5.04	0.0393
glucose	1	16	0.09	0.7669

Group	Group	Estimate	P-value
DM-Delayed	DM-Normal	-1.1420	0.0393

Analysis of endpoint Lastnum by group - Diabetics only
Adjusted for Insulin
Overall and between group tests based on fitted model

Type 3 Tests of Fixed Effects

Effect	Num DF	Den DF	F Value	Pr > F
Group	1	17	7.01	0.0169
Insulin	1	17	0.24	0.6313

Group	Group	Estimate	P-value
DM-Delayed	DM-Normal	-1.3204	0.0169

Analysis of endpoint Lastnum by group - Diabetics only
Adjusted for Oral_hypo
Overall and between group tests based on fitted model

Type 3 Tests of Fixed Effects

Effect	Num DF	Den DF	F Value	Pr > F
Group	1	17	6.47	0.0210
Oral_hypo	1	17	0.40	0.5373

Group	Group	Estimate	P-value
DM-Delayed	DM-Normal	-1.2268	0.0210

Analysis of endpoint Lastnum by group - Diabetics only
Adjusted for Antidep
Overall and between group tests based on fitted model

Type 3 Tests of Fixed Effects

Effect	Num DF	Den DF	F Value	Pr > F
Group	1	17	6.76	0.0186
Antidep	1	17	1.96	0.1799

Group	Group	Estimate	P-value
DM-Delayed	DM-Normal	-1.2003	0.0186
